# Potentials of bone marrow cells-derived from naïve or diabetic mice in autoimmune type 1 diabetes: immunomodulatory, anti-inflammatory, anti hyperglycemic, and antioxidative

**DOI:** 10.1007/s12020-024-03929-7

**Published:** 2024-07-17

**Authors:** Soha Gomaa, Mohamed Nassef, Amira Hafez

**Affiliations:** Zoology Department, Faculty of Science, Tanta University, Tanta, Egypt

**Keywords:** Adoptive cell transfer, Anti-diabetic, Anti-inflammatory, Anti-oxidant defense, Bone marrow cells, Diabetes mellitus

## Abstract

**Background:**

The scarcity of transplanted human islet tissue and the requirement for immunosuppressive drugs to prevent the rejection of allogeneic grafts have hindered the treatment of autoimmune type 1 diabetes mellitus (T1DM) through islet transplantation. However, there is hope in adoptively transferred bone marrow cells (BMCs) therapy, which has emerged as a propitious pathway for forthcoming medications. BMCs have the potential to significantly impact both replacement and regenerative therapies for a range of disorders, including diabetes mellitus, and have demonstrated anti-diabetic effects.

**Aim:**

The main goal of this study is to evaluate the effectiveness of adoptively transferred bone marrow cells derived from either naïve mice (nBMCs) or diabetic mice (dBMCs) in treating a T1DM mice model.

**Methods:**

Male Swiss albino mice were starved for 16 h and then injected with streptozotocin (STZ) at a dose of 40 mg/kg body weight for 5 consecutive days to induce T1DM. After 14 days, the diabetic mice were distributed into four groups. The first group served as a diabetic control treated with sodium citrate buffer, while the other three groups were treated for two weeks, respectively, with insulin (subcutaneously at a dose of 8 U/kg/day), nBMCs (intravenously at a dose of 1 × 10^6^ cells/mouse/once), and dBMCs (intravenously at a dose of 1 × 10^6^ cells/mouse/once).

**Results:**

It is worth noting that administering adoptively transferred nBMCs or adoptively transferred dBMCs to STZ-induced T1DM mice resulted in a significant amelioration in glycemic condition, accompanied by a considerable reduction in the level of blood glucose and glycosylated hemoglobin % (HbA1C %), ultimately restoring serum insulin levels to their initial state in control mice. Administering nBMCs or dBMCs to STZ-induced T1DM mice led to a remarkable decrease in levels of inflammatory cytokine markers in the serum, including interferon-γ (INF-γ), tumor necrosis factor- α (TNF-α), tumor growth factor-β (TGF-β), interleukin-1 β (L-1β), interlekin-4 (IL-4), interleukin-6 (IL-6), and interleukin-10 (IL-10). Additionally, STZ-induced T1DM mice, when treated with nBMCs or dBMCs, experienced a notable rise in total immunoglobulin (Ig) level. Furthermore, there was a significant reduction in the levels of islet cell autoantibodies (ICA) and insulin autoantibodies (IAA). Furthermore, the serum of STZ-induced T1DM mice showed a significant increase in Zinc transporter 8 antigen protein (ZnT8), islet antigen 2 protein (IA-2), and glutamic acid decarboxylase antigen protein (GAD) levels. Interestingly, the administration of nBMCs or dBMCs resulted in a heightened expression of IA-2 protein in STZ-induced T1DM mice treated with nBMCs or dBMCs. Furthermore, the level of malondialdehyde (MDA) was increased, while the levels of catalase (CAT) and superoxide dismutase (SOD) were decreased in non-treated STZ-induced T1DM mice. However, when nBMCs or dBMCs were administered to STZ-induced T1DM mice, it had a significant impact on reducing oxidative stress. This was accomplished by reducing the levels of MDA in the serum and enhancing the activities of enzymatic antioxidants like CAT and SOD. STZ-induced T1DM mice displayed a significant elevation in the levels of liver enzymes ALT and AST, as well as heightened levels of creatinine and urea. Considering the crucial roles of the liver and kidney in metabolism and excretion, this research further examined the effects of administering nBMCs or dBMCs to STZ-induced T1DM mice. Notably, the administration of these cells alleviated the observed effects.

**Conclusion:**

The present study suggests that utilizing adoptively transferred nBMCs or adoptively transferred dBMCs in the treatment of T1DM led to noteworthy decreases in blood glucose levels, possibly attributed to their capacity to enhance insulin secretion and improve the performance of pancreatic islets. Additionally, BMCs may exert their beneficial effects on the pancreatic islets of diabetic mice through their immunomodulatory, antioxidant, anti-inflammatory, and anti-oxidative stress properties.

## Introdcution

Type 1 diabetes mellitus (T1DM) is a medical disorder resulting from an autoimmune disease, wherein pancreatic β-cells are destroyed by autoreactive T cells targeting and attack, leading to insulitis and a total absence of insulin secretion [1]. The exact cause of the immune disorder in T1DM is still unknown, but it is believed to be a combination of genetic susceptibility and environmental factors [2]. The available treatment choices for T1DM are restricted and heavily depend on insulin replacement therapy for the regulation of glycemic levels [3]. However, this treatment can solely serve as a supplement for the deficient insulin and is incapable of reinstating the pancreas or β-cell’s functionality [4]. Insulin injections and dietary control are important approaches of managing T1DM, but they cannot fully replicate the body’s natural ability to produce insulin, which can lead to severe complications. In some cases, insulin therapy may not be sufficient in managing the diabetes progression and its associated complications [5, 6]. Therefore, alternative therapeutic approaches, such as pancreas or pancreatic islet cell transplantation, have been explored. However, these approaches are limited by donor scarcity and rejection complications. The most desirable approach would be to enhance the renewal of the body’s own β-cells [7, 8]. Bone marrow transplantation (BMT) has been a focus of research in recent years for T1DM [9].

BMT offers an effective solution for treating T1DM by harnessing the immunomodulatory advantages of bone marrow-derived cells (BMCs) and their potential to differentiate into insulin-producing cells. This is particularly important as T1DM is an autoimmune disorder that triggers the immune system attacking and destroying pancreatic β-cells. Although BMT medication has proven to be effective in controlling blood glucose levels and minimizing major fluctuations, it does not possess the ability to entirely avert the occurrence of complications such as microvascular, macrovascular, and neuropathy complications. Consequently, the generation of new β-cells remains a critical component of T1DM treatment [10, 11].

BMT-based therapy possesses the potential to contribute to the renewal of β-cells. It plays a crucial role in the development of various cell lineages, including endothelial precursor cells that help in microvascular angiogenesis [12]. This is particularly significant for β-cell renewal since the pancreatic vascular endothelium can stimulate β-cell differentiation from progenitors and provide essential signals like hepatocyte growth factor (HGF) to stimulate β-cell function and proliferation [13].

BMT has been shown to activate various mechanisms that improve islet cell function in patients with T1DM, including the secretion of cytokines and growth factors, induction of angiogenesis and vascularization, and the endogenous stem cells proliferation [14]. BMT has also been known to ameliorate islet cell function by eliminating autoreactive T cells and producing a less inflammatory environment [15]. Bone marrow (BM) therapy for T1DM is more efficient due to its strong immune-regulating capability [16, 17]. BMT have the unique ability to transdifferentiate into β-cells-like organoids, leading to an increase in islet mass. Moreover, they display immunomodulatory characteristics by suppressing the immune response of T and Th1 cells through inflammatory and tumor growth factor- β (TGF-β) pathways, which is principally applicable in T1DM [11, 18–20]. BMT, which has the efficacy to discriminate into β-cell progenitors and exhibit immunotolerance, holds promise as a T1DM treatment. Research has indicated that the BMT exhibits significant efficacy in enhancing insulin sensitivity, regulating insulin levels, and managing hyperglycemia. Furthermore, BMT medications have demonstrated potential in reverting autoimmunity and enhancing blood glucose control in T1DM [21].

Research in regenerative medicine for diabetes treatment has identified BMCs as a promising candidate for generating pancreatic β-cells. Lechner, Habener [22]. Although there is debate over whether BMCs can stimulate β-cell differentiation or transdifferentiate into β-cells, studies have shown that BMCs can directly transdifferentiate into β-cells. BMT has been found to enhance hyperglycemia in diabetic models and improve β-cell regeneration after streptozotocin (STZ)-induced diabetic damage. The accumulation of BMCs around the islets in STZ-BMT mice approved that they may play a crucial role in initiating the differentiation and proliferation of stem/progenitor cells into β-cells [23]. In addition to regulating blood glucose levels, the managing of immune disorders is also crucial in controlling T1DM. BMCs possess immunomodulatory potentials through numerous mechanisms, including interaction with CD4 + T and CD8 + T cells through molecular program death ligand 1 (PD-L1), nitric oxide (NO) secretion, and suppression of mitogen activity to control lymphocyte proliferation [24, 25]. These immunomoregulation approaches of BMCs suggest that they can suppress the proliferation of specific T cells, such as Glutamic Acid Decarboxylase (GAD)-specific T cells. BMT has shown promising results in modulating the immune system, decreasing inflammation, and elevating T cell regulation, which is essential for the restoration of helper cells type 1 and type 2 (Th1 and Th2), cytokine balance, and local immune modulation to protect pancreatic islet β cells from lymphocyte-mediated damage [26–28]. Furthermore, BMT can stimulate chemokines secretion, which can potentially revert antibody secretion and contribute to the overall management of T1DM [29, 30].

BMT possess antioxidant and anti-inflammatory features, making them valuable in the mitigation of tissue necrosis. These cells have the potential to augment the body’s antioxidant defense system and decrease oxidative stress, thereby potentially preventing the development of diabetes-related complications [31, 32]. Animal models of diabetes have shown that BMT can improve glycemic control and reduce inflammation, making them a promising therapeutic option for treating diabetes and its associated complications [33]. Insulinoma-associated antigen 2 (IA-2) and zinc transporter protein member 8 (ZnT8) are two important autoantigens involved in the pathogenesis of type 1 diabetes mellitus (T1DM). The absence of IA-2 and ZnT8 leads to a decrease in insulin content and secretion [34]. IA-2 and GAD have a critical role in maintaining glucose hemostasis and are targeted by autoantibodies in 60-80% of new-onset D1TM patients [35]. Autoantibodies against pancreatic islet cells (ICA), IA-2, insulin, and GAD are highly specific in autoimmune diabetes and are characteristic of the first stage of T1DM and predictive of future diabetes onset [36, 37]. Autologous BMT, devoid of immunoablation, has demonstrated efficacy and safety as a mechanism to significantly reduce the generation and influence of autoantibodies against ICA, IA-2, GAD, insulin and inhibiting autoimmune attack and pancreatic damage, which emphasizes the potential of BMT as a therapeutic mechanism for D1TM [9, 11, 28, 38, 39].

The purpose of this work is to evaluate the therapeutic potential of adoptively transferred BMCs from either naïve (nBMCs) or diabetic mice (dBMCs), as well as their capacity to ameliorate the symptoms associated with T1DM in a mouse model with STZ-induced D1TM.

## Materials and methods

### Antibodies and reagents

Streptozotocin (STZ) from Sigma Aldrich, UK, was utilized to induce diabetes in mice. It was dissolved in 0.1 M Na-citrate buffer. Roswell Park Memorial Institute medium 1640 (RPMI-1640) was obtained from Invitrogen, USA. The medium was enriched with 10% v/v heat-inactivated fetal bovine serum (FBS), streptomycin/penicillin (100 IU/ml), 2-mM L-glutamine, 1-mM sodium pyruvate, and non-essential amino acids. Ammonium chloride potassium (ACK) lysis buffer was purchased from Lonza, Bio Whittaker, USA. Monoclonal antibodies (mAbs) including anti-mouse CD4, anti-mouse CD8, anti-mouse NKp46 (CD335), anti-mouse CD11b, anti-mouse Ly6g, anti-mouse CD25, anti-mouse FOXP3, annexin V, and PI were obtained from eBioscience, San Diego, CA, USA.

### Mice

A total of 50 male Swiss albino mice, aged 6-8 weeks and weighing 25 ± 2 g, were procured from the National Research Centre Animal House in Dokki, Giza, Egypt. These mice were then distributed into 5 groups of 10 and provided with a standard pellet diet and tap water ad libitum. The experimental procedure was conducted following the guidelines of the Institutional Animal Care and Use Committee (IACUC) approved by Faculty of Science, Tanta University, Tanta, Egypt. Throughout the study, we diligently followed the ARRIVE guidelines to guarantee the animals were provided with optimal care. All procedures were conducted ethically and humanely.

### Induction of type 1 diabetes

Diabetes was induced in experimental mice through the administration of STZ via intraperitoneal (i.p.) injection at a dosage of 40 mg/kg body weight. The STZ was dissolved in a freshly prepared 0.1 M sodium citrate buffer with a pH of 4.5 and administered to the mice for a duration of 5 consecutive days. To prevent hypoglycemia caused by STZ, the mice were allowed to drink a glucose solution overnight, which had a concentration of 10% w/v. Control mice received only the citrate buffer. After the dosing was completed, the STZ-treated mice were kept under normal conditions for 9 days, during which they developed diabetes, as indicated by fasting blood glucose levels of ≥ 250 mg/dl. Following the experiment, the STZ-treated mice were subjected to a 12-hour fast. Blood samples were then taken from their tail veins to measure their blood glucose levels. Mice in the diabetic groups, with fasting blood glucose levels exceeding 250 mg/dl, were selected for further studies.

### Preparation of bone marrow cells

To perform adoptive transfer of bone marrow (BM) cells, unfractionated BM cells were collected from either naïve or STZ-induced T1DM mice and processed to obtain a single cell suspension [40]. The freshly harvested BM cells were washed and resuspended in PBS, treated with ACK buffer to lyse red blood cells, and washed again before being resuspended in RPMI 1640 supplemented with 5% fetal calf serum (FCS). The cell count was determined using the Trypan blue dye exclusion assay in a Neubauer hemocytometer. Finally, the BM cells were transferred into STZ-induced T1DM mice via the lateral tail vein at a concentration of 1 × 10^6^ cells per mouse.

### In vivo study design

**Fig. 1:**
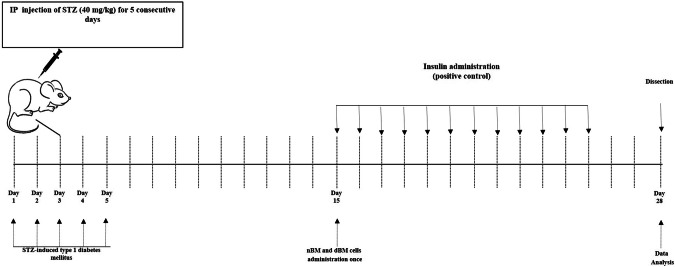
Induction of type 1 diabetes and in vivo study design

Figure 1 illustrates the induction of diabetes and the design of the in vivo study. STZ-induced T1DM mice were randomly divided into four groups, each consisting of 10 mice. On day 15 after STZ injection, the first group (referred to as the STZ group) i.p. administrated sodium citrate buffer. The second group (referred to as the STZ/Insulin group) was subcutaneously (s.c.) injected with insulin at a dosage of 8 U/kg/day. The third group (referred to as the STZ/nBMC group) received intravenous (i.v.) injection of BM cells derived from naïve mice, with a dosage of 1 × 10^6^ cells/mouse, administered once. The fourth group (referred to as the STZ/dBMC group) received i.v. injection of BM cells derived from STZ-induced T1DM mice, with a dosage of 1 × 10^6^ cells/mouse, administered once. Additionally, there was a group of naïve mice that received i.p. administration of sodium citrate buffer alone. On day 28 after STZ injection (day 14 after treatment), all groups of mice were sacrificed, and their spleens and sera were collected for testing the antidiabetic effectiveness, immunomodulatory potencies, and biochemical examinations.

### Biochemical analyses

Blood glucose level (mg/dl) was estimated in all experimental animals pre- and post-administration of STZ. For STZ-induced D1TM mice, blood glucose levels were routinely measured until reaching diabetes induction (mice with blood glucose ≥ 250 mg/dl are indicted as diabetics). Serum insulin level was estimated using a mice insulin ELISA kit. Glycosylated hemoglobin (HbA1c) was determined using ELISA kits (MyBiosource, San Diego, CA, USA). All assays were conducted in accordance with the guidelines provided with the commercial kits.

### Splenocytes harvesting and counting

The preparation and quantification of splenocytes were carried out in accordance with the methods outlined by Gomaa [41] and Nassef [42] and. briefly, mice were sacrificed and their spleens were extracted and placed in PBS. Subsequently, the splenocytes were suspended in ACK buffer to lyse erythrocytes, washed, and resuspended in PBS. Finally, the cells were enumerated using a Trypan blue dye exclusion assay in a Neubauer hemocytometer.

### Analyses of oxidative stress and antioxidants

Serum levels of superoxide dismutase (SOD), malondialdehyde (MDA) and catalase (CAT) were measured using the thiobarbituric acid method, xanthine oxidase, and the ammonium molybdate colorimetric method, respectively (Ohkawa et al., 1979; Misra and Fridovich, 1972; Aebi, 1984). The assays were performed following the instructions provided with the commercial kits.

### Assessment of anti-inflammatory cytokines, total antibodies and autoantibodies

The serum levels of interleukin-1 β I(L-1β), interlekin-4 (IL-4), interleukin-6 (IL-6), interleukin-10 (IL-10), tumor necrosis factor- α (TNF-α), interferon-γ (INF-γ), tumor growth factor-β (TGF-β) and the total immunoglobulin (Ig) were assessed by enzyme-linked immunosorbent assay (ELISA) with a commercially available kit (BioLegend, San Diego, USA). The concentrations of autoantibodies against glutamate decarboxylase (GADA), autoantibodies against zinc transporter 8 (ZnT8A), autoantibodies against islet cell antibodies (ICA), autoantibodies against insulin (IAA) and autoantibodies against Insulinoma associated antigen 2 (IA-2A) were measured in pancreatic tissue by Sandwich Enzyme Immuno-Assay (EIA). All appropriate controls and standards, as specified by the manufacturer’s kit, were used.

### Western Blot analysis of insulinoma associated antigen 2

The Western Blot analysis of the isolated pancreatic protein (Bio-Rad, Inc., USA) was conducted using the anti- insulinoma-associated protein 2 (INSM2) mouse antibody recognizing INSM2 using INSM2 antibody kit (GeneTex, Inc., USA). All experimental protocols were executed in compliance with the guidelines stipulated by the manufacturers. To begin, the pancreatic proteins were electrophoretically transferred onto a Hybond ECL nitrocellulose membrane (Amersham, U.K.) at 100 V in a solution containing 48 mmol/l Tris, 39 mmol/l glycine (pH 9.2), and 20% (v/v) methanol for a duration of 90 min. Subsequently, the membrane was blocked overnight at 4 °C using a blocking solution composed of 5% (wt/vol) fat-free dry milk in phosphate-buffered saline. Following the blocking step, the membrane was incubated for 1 h at room temperature with the anti-INSM2 mouse antibody, which was diluted in the blocking solution at a ratio of 1:1,000. Afterward, the membrane was washed three times with PBS-T and then blocked for 30 min using the blocking solution. Next, the membrane was incubated for 1 h at room temperature with the anti-IgG horseradish-peroxidase conjugate. Following another round of washing with PBS-T, the bands on the membrane were visualized using the SuperSignal West Pico chemiluminescent substrate (Pierce, Rockford, IL, USA). The chemiluminescence signal was detected through autoradiography. To determine the intensity of the target proteins, image analysis software was employed. The intensity of the target proteins’ band was normalized against housekeeping beta actin protein utilizing protein normalization on the ChemiDoc MP imager.

### Analysis of liver and kidney functions

The serum alanine aminotransferase (ALT), aspartate aminotransferase (AST), creatinine, and urea levels were assessed using a fully-automatic biochemical analyzer (Vita lab Selectra E, German) and a standard commercial kit (BIOLABO SAS, Les Hautes Rives, 02160, Maizy, France). The evaluation was performed colorimetrically. The experiment strictly adhered to the manufacturer’s manuals.

### Leucocytes analysis

Mice were anesthetized using a mild ether solution. Blood samples were then collected from their retro-orbital plexus using microhematocrit tubes that had been treated with heparin. The collected blood samples were analyzed using automated hematology analyzer (model MEK-6318K, Japan) to determine various blood parameters, such as the lecocyte total count (10^3^/cmm) and their differential relative percent (neutrophils, lymphocytes, basophils, and monocytes) in peripheral blood (PB).

### Statistical analysis

The results were represented as mean ± SE. Statistical analyses was carried out by one-way analysis of variance (ANOVA) followed by *post hoc* Tukey HSD’s test and Dunnett’s test and p value < 0.05 were regarded as significant.

## Results

### Antidiabetic potentials of adoptively transferred BMCs

**Fig. 2:**
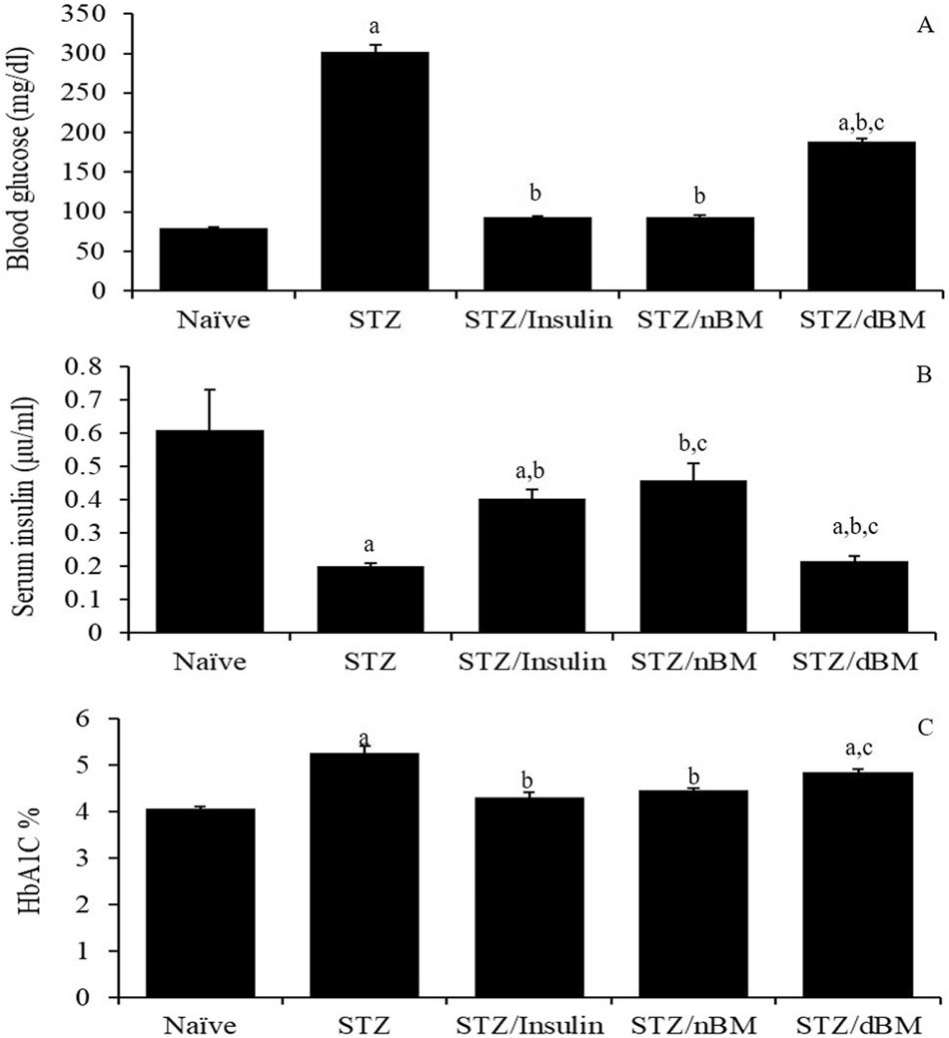
Antidiabetic potentials of bone marrow cells in STZ-induced T1DM mice treated with adaptively transferred bone marrow-derived cells. **A** Blood glucose level in naïve, STZ-induced T1DM mice treated with sodium citrate buffer, STZ-induced T1DM mice treated with insulin, STZ-induced T1DM mice treated with nBMCs and STZ-induced T1DM mice treated with dBMCs. **B** Serum insulin level in naïve, STZ-induced T1DM mice treated with sodium citrate buffer, STZ-induced T1DM mice treated with insulin, STZ-induced T1DM mice treated with nBMCs and STZ-induced T1DM mice treated with dBMCs. **C** HbA1C % in naïve, STZ-induced T1DM mice treated with sodium citrate buffer, STZ-induced T1DM mice treated with insulin, STZ-induced T1DM mice treated with nBMCs and STZ-induced T1DM mice treated with dBMCs. Data were expressed as mean ± SE. Statistical significant was considered as *P* value less than 0.05. Note: ^a, b, c^ Statistically significant difference as compared to the corresponding means of the naive group (a), STZ-induced T1DM mice treated with sodium citrate buffer (b), STZ-induced T1DM mice treated with insulin (c) within each column

The results here indicated that the treatment of STZ-induced T1DM mice with sodium citrate buffer or adoptively transferred dBMCs significantly increased the blood glucose level comparing to that in naïve mice received sodium citrate buffer (302.25 ± 8.90 mg/dl and 188.25 ± 3.88 mg/dl, respectively versus 79.75 ± 1.5 mg/dl) (Fig. 2A). The administration of STZ-induced T1DM mice with insulin, adoptively transferred nBMCs or adoptively transferred dBMCs significantly reduced the blood glucose levels comparing to that in STZ-induced T1DM mice treated with sodium citrate buffer (93.00 ± 1.82 mg/dl, 92.75 ± 2.52 mg/dl and 188.25 ± 3.88 mg/dl, respectively versus 302.25 ± 8.90 mg/dl), however administration of STZ-induced T1DM mice with adoptively transferred dBMCs significantly increased the blood glucose levels comparing to that in STZ-induced T1DM mice treated with insulin (188.25 ± 3.88 mg/dl versus 93.00 ± 1.82 mg/dl) (Fig. 2A). Furthermore, there were significant increases in the level of serum insulin post administration of insulin, adoptively transferred nBMCs and adoptively transferred dBMCs comparing to that in STZ-induced T1DM mice treated with sodium citrate buffer (0.41 ± 0.4 µU/ml, 0.46 ± 0.07 µU/ml and 0.22 ± 0.02 µU/ml, respectively versus 0.20 ± 0.01 µU/ml) (Fig. 2B).

On the other hand, the administration of STZ-induced T1DM mice treated with sodium citrate buffer, insulin, adoptively transferred nBMCs and adoptively transferred dBMCs significantly decreased the level of serum insulin comparable to naïve mice received sodium citrate buffer (0.20 ± 0.01 µU/ml, 0.41 ± 0.40 µU/ml, 0.46 ± 0.07 µU/ml and 0.22 ± 0.02 µU/ml, respectively versus 0.61 ± 0.17 µU/ml) (Fig. 2B). The administration of STZ-induced T1DM mice with adoptively transferred nBMCs significantly increased the level of serum insulin, while their administration with adoptively transferred dBMCs significantly decreased the level of serum insulin when compared to that in STZ-induced T1DM mice received insulin (0.46 ± 0.07 µU/ml and 0.22 ± 0.02 µU/ml, respectively versus 0.41 ± 0.4 µU/ml) (Fig. 2B).

Additionally, the results showed that the administration of STZ-induced T1DM mice with sodium citrate buffer, insulin, adoptively transferred nBMCs and adoptively transferred dBMCs significantly decreased the percentage of glycosylated hemoglobin (HbA1C) comparing to that in naïve mice received sodium citrate buffer (5.25 ± 0.15 and 4.30 ± 0.10%, 4.50 ± 0.05 and 4.9 ± 0.05%, respectively versus 4.10 ± 0.05% (Fig. 2C). On the other hand, treatment of STZ-induced T1DM mice with insulin and adoptively transferred nBMCs significantly decreased the percentage of serum HbA1C comparing to that in STZ-induced T1DM mice received sodium citrate buffer alone (4.30 ± 0.10 and 4.50 ± 0.05%, respectively versus 5.25 ± 0.15%), while the treatment of STZ-induced T1DM mice with adoptively transferred dBMCs significantly increased the level of serum HbA1C% comparing to that in STZ-induced T1DM mice received insulin (4.9 ± 0.05 versus 4.30 ± 0.10%) (Fig. 2C).

### Cellularity effect of adoptively transferred BMCs

**Fig. 3:**
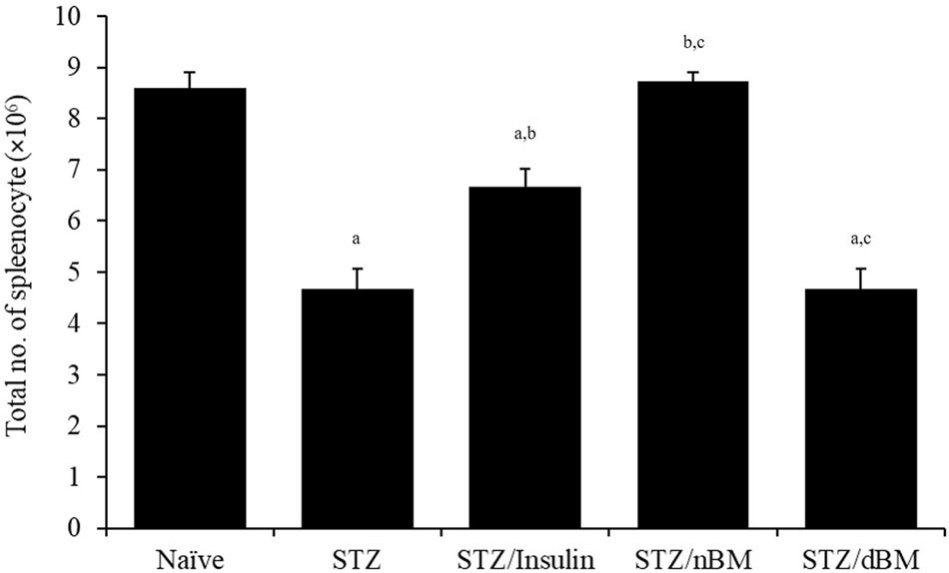
Cellularity effect of bone marrow cells in STZ-induced T1DM mice treated with adaptively transferred bone marrow-derived cells. STZ-induced T1DM mice treated with sodium citrate buffer, STZ-induced T1DM mice treated with insulin, STZ-induced T1DM mice treated with nBMCs and STZ-induced T1DM mice treated with dBMCs. Data were expressed as mean ± SE. Statistical significant was considered as *P* value less than 0.05. Note: ^a,b,c^ Statistically significant difference as compared to the corresponding means of the naive group (a), STZ-induced T1DM mice treated with sodium citrate buffer (b), STZ-induced T1DM mice treated with insulin(c) within each column

The current study revealed that administration of STZ-induced T1DM mice with sodium citrate buffer, insulin and adoptively transferred dBMCs noticeably decreased the total number of splenocytes raised the number of splenocytes comparing to naïve mice received sodium citrate buffer (4.70 ± 0.40 × 10^6^, 6.70 ± 0.40 × 10^6^ and 4.70 ± 0.40 × 10^6^, respectively versus 8.60 ± 0.30 × 10^6^) (Fig. 3). On the other hand, the treatment of STZ-induced T1DM mice with insulin or adoptively transferred nBMCs significantly increased the total splenocytes count comparing to that in STZ-induced T1DM mice treated with sodium citrate buffer (6.70 ± 0.40 × 10^6^ and 8.70 ± 0.20 × 10^6^, respectively versus 4.70 ± 0.40 × 10^6^) (Fig. 3). Admiration of STZ-induced T1DM mice with adoptively transferred nBMCs significantly increased the total number of splenocytes, while their administration with adoptively transferred dBMCs significantly decreased the splenocytes count comparing to that in STZ-induced T1DM mice treated with insulin (8.70 ± 0.20 × 10^6^ and 4.70 ± 0.40 × 10^6^, respectively versus 6.70 ± 0.40 × 10^6^) (Fig. 3).

### The potential of adoptively transferred BMCs against diabetic-related pro-and anti-inflammatory cytokines

**Fig. 4:**
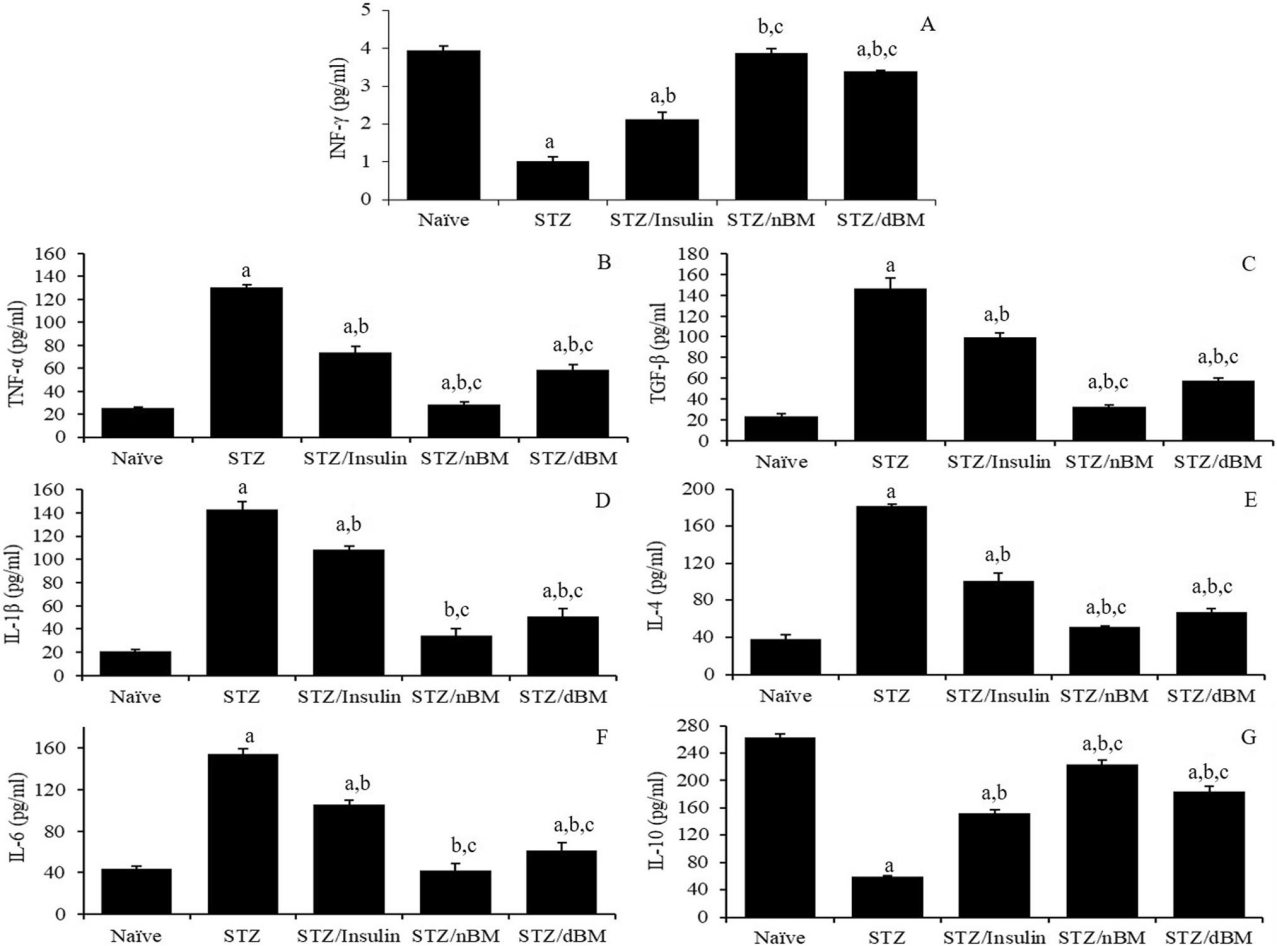
The potentials of bone marrow cells against diabetic-related pro-and anti-inflammatory cytokines in STZ-induced T1DM mice treated with adaptively transferred bone marrow-derived cells. **A** INF-γ level in naïve, STZ-induced T1DM mice treated with sodium citrate buffer, STZ-induced T1DM mice treated with insulin, STZ-induced T1DM mice treated with nBMCs and STZ-induced T1DM mice treated with dBMCs. **B** TNF-α level in naïve, STZ-induced T1DM mice treated with sodium citrate buffer, STZ-induced T1DM mice treated with insulin, STZ-induced T1DM mice treated with nBMCs and STZ-induced T1DM mice treated with dBMCs. **C** TGF-β level in naïve, STZ-induced T1DM mice treated with sodium citrate buffer, STZ-induced T1DM mice treated with insulin, STZ-induced T1DM mice treated with nBMCs and STZ-induced T1DM mice treated with dBMCs. **D** IL-1β level in naïve, STZ-induced T1DM mice treated with sodium citrate buffer, STZ-induced T1DM mice treated with insulin, STZ-induced T1DM mice treated with nBMCs and STZ-induced T1DM mice treated with dBMCs. **E** IL-4 level in naïve, STZ-induced T1DM mice treated with sodium citrate buffer, STZ-induced T1DM mice treated with insulin, STZ-induced T1DM mice treated with nBMCs and STZ-induced T1DM mice treated with dBMCs. **F** IL-6 level in naïve, STZ-induced T1DM mice treated with sodium citrate buffer, STZ-induced T1DM mice treated with insulin, STZ-induced T1DM mice treated with nBMCs and STZ-induced T1DM mice treated with dBMCs. **G** IL-10 level in naïve, STZ-induced T1DM mice treated with sodium citrate buffer, STZ-induced T1DM mice treated with insulin, STZ-induced T1DM mice treated with nBMCs and STZ-induced T1DM mice treated with dBMCs. Data were expressed as mean ± SE. Statistical significant was considered as *P* value less than 0.05. Note: ^a,b,c^ Statistically significant difference as compared to the corresponding means of the naive group (a), STZ-induced T1DM mice treated with sodium citrate buffer (b), STZ-induced T1DM mice treated with insulin(c) within each column

Our data revealed that the administration of STZ-induced T1DM mice with sodium citrate buffer, insulin and adoptively transferred dBMCs significantly decreased the serum level of INF-γ comparing to that in naive mice received sodium citrate buffer (1.01 ± 0.11 pg/ml, 2.11 ± 0.20 pg/ml, 3.88 ± 0.11 pg/ml and 3.38 ± 0.03 pg/ml, respectively versus 3.94 ± 0.13 pg/ml) (Fig. 4A). On the other hand, the treatment of STZ-induced T1DM mice with insulin, adoptively transferred nBMCs and adoptively transferred dBMCs significantly increased the serum level of INF-γ comparing to that in STZ-induced T1DM mice received sodium citrate buffer (2.11 ± 0.20 pg/ml, 3.88 ± 0.11 pg/ml and 3.38 ± 0.06 pg/ml, respectively versus 1.02 ± 0.11 pg/ml) (Fig. 4A). The administration of STZ-induced T1DM mice with adoptively transferred nBMCs and adoptively transferred dBMCs significantly decreased the serum level of INF-γ comparing to that in STZ-induced T1DM mice treated with insulin (3.88 ± 0.11 pg/ml and 3.38 ± 0.06 pg/ml, respectively versus 2.13 ± 0.35 pg/ml) (Fig. 4A).

Our results indicated that the administration of STZ-induced T1DM mice with sodium citrate buffer, insulin and adoptively transferred dBMCs significantly increased the serum level of TNF-α comparing to that in naive mice received sodium citrate buffer (130.47 ± 2.21 pg/ml, 73.50 ± 5.42 pg/ml, 28.73 ± 1.61 pg/ml and 58.80 ± 4.06 pg/ml, respectively versus 25.07 ± 0.78 pg/ml) (Fig. 4B). Furthermore, the injection of STZ-induced T1DM mice with insulin, adoptively transferred nBMCs and adoptively transferred dBMCs significantly decreased the serum level of TNF-α comparing to that in STZ-induced T1DM mice received sodium citrate buffer (73.50 ± 5.42 pg/ml, 28.73 ± 1.61 pg/ml and 58.80 ± 4.06 pg/ml, respectively versus 130.47 ± 2.21 pg/ml) (Fig. 4B). The administration of STZ-induced T1DM mice with adoptively transferred nBMCs and adoptively transferred dBMCs significantly decreased the serum level of TNF-α comparing to that in STZ-induced T1DM mice treated with insulin (28.73 ± 1.61 pg/ml and 58.80 ± 4.06 pg/ml, respectively versus 73.50 ± 5.42 pg/ml) (Fig. 4B).

The data here revealed that the treatment of STZ-induced T1DM mice with sodium citrate buffer, insulin and adoptively transferred dBMCs significantly increased the serum level of TGF-β comparing to that in naive mice received sodium citrate buffer (146.67 ± 9.56 pg/ml, 99.47 ± 3.98 pg/ml, 32.53 ± 2.00 pg/ml and 27.96 ± 2.40 pg/ml, respectively versus 23.43 ± 2.10 pg/ml) (Fig. 4C). Moreover, the administration of STZ-induced T1DM mice with insulin, adoptively transferred nBMCs and adoptively transferred dBMCs significantly decreased the serum level of TGF-β comparing to that in STZ-induced T1DM mice received sodium citrate buffer (99.47 ± 3.98 pg/ml, 32.53 ± 2.00 pg/ml and 27.96 ± 2.40 pg/ml, respectively versus 146.67 ± 9.56 pg/ml) (Fig. 4C). The treatment of STZ-induced T1DM mice with adoptively transferred nBMCs or adoptively transferred dBMCs significantly decreased the serum level of TGF-β comparing to that in STZ-induced T1DM mice treated with insulin (32.53 ± 2.00 pg/ml and 27.96 ± 2.40 pg/ml, respectively versus 99.47 ± 3.98 pg/ml) (Fig. 4C).

The treatment of STZ-induced T1DM mice with insulin, adoptively transferred nBMCs and adoptively transferred dBMCs significantly reduced the serum level of IL-1β comparable to that in STZ-induced T1DM mice treated with sodium citrate buffer (108.77 ± 2.3 pg/ml, 34.30 ± 5.98 pg/ml and 50.60 ± 6.80 pg/ml, respectively versus 142.87 ± 6.50 pg/ml) (Fig. 4D). Moreover, the administration of STZ-induced T1DM mice with sodium citrate buffer, insulin and adoptively transferred dBMCs significantly increased the serum level of IL-1β comparing to that in naive mice received sodium citrate buffer (142.87 ± 6.50 pg/ml, 108.77 ± 2.38 pg/ml and 50.60 ± 6.80 pg/ml, respectively versus 21.10 ± 1.50 pg/ml) (Fig. 4D). The treatment of STZ-induced T1DM mice with adoptively transferred nBMCs or adoptively transferred dBMCs significantly reduced the serum level of IL-1β comparing to that in STZ-induced T1DM mice treated with insulin (34.30 ± 5.98 pg/ml and 50.60 ± 6.80 pg/ml, respectively versus 108.77 ± 2.38 pg/ml) (Fig. 4D).

Our results showed that the treatment of STZ-induced T1DM mice with insulin, adoptively transferred nBMCs and adoptively transferred dBMCs significantly reduced the level of serum IL-4 comparing to that in STZ-induced T1DM mice received sodium citrate buffer (101.37 ± 7.88 pg/ml, 51.00 ± 1.59 pg/ml and 67.17 ± 3.67 pg/ml, respectively versus 181.47 ± 2.54 pg/ml) (Fig. 4E). Moreover, the treatment of STZ-induced T1DM mice with sodium citrate buffer, insulin, adoptively transferred nBMCs and adoptively transferred dBMCs significantly increased the serum level of IL-4 comparing to that in naive mice received sodium citrate buffer (181.47 ± 2.54 pg/ml, 101.37 ± 7.88 pg/ml, 51.00 ± 1.59 pg/ml and 67.17 ± 3.67 pg/ml respectively versus 38.60 ± 3.85 pg/ml) (Fig. 4E). The treatment of STZ-induced T1DM mice with adoptively transferred nBMCs or adoptively transferred dBMCs significantly decreased the serum level of IL-4 comparing to that in STZ-induced T1DM mice treated with insulin (51.00 ± 1.59 pg/ml and 67.17 ± 3.67 pg/ml, respectively versus 101.37 ± 7.88 pg/ml) (Fig. 4E).

The inoculation of STZ-induced T1DM mice with insulin, adoptively transferred nBMCs and adoptively transferred dBMCs significantly decreased the serum level of IL-6 comparing to that in STZ-induced T1DM mice received sodium citrate buffer (105.77 ± 4.05 pg/ml, 41.83 ± 6.96 pg/ml and 61.63 ± 7.35 pg/ml, respectively versus 153.93 ± 5.08 pg/ml) (Fig. 4F). Moreover, the injection of STZ-induced T1DM mice with sodium citrate buffer, insulin and adoptively transferred dBMCs significantly decreased the serum level of IL-6 comparing to that in naive mice received sodium citrate buffer (153.93 ± 5.08 pg/ml, 105.77 ± 4.05 pg/ml and 61.63 ± 7.35 pg/ml, respectively versus 43.47 ± 2.60 pg/ml) (Fig. 4F). The injection of STZ-induced T1DM mice with adoptively transferred nBMCs or adoptively transferred dBMCs significantly decreased the serum level of IL-6 comparing to that in STZ-induced T1DM mice treated with insulin (41.83 ± 6.96 pg/ml and 61.63 ± 7.35 pg/ml, respectively versus 105.77 ± 4.05 pg/ml) (Fig. 4F).

The treatment of STZ-induced T1DM mice with insulin, adoptively transferred nBMCs and adoptively transferred dBMCs markedly increased the serum level of IL-10 comparing to that in STZ-induced T1DM mice received sodium citrate buffer (152.33 ± 5.05 pg/ml, 223.13 ± 6.75 pg/ml and 183.93 ± 7.34 pg/ml, respectively versus 59.00 ± 1.39 pg/ml) (Fig. 4G). Furthermore, the treatment of STZ-induced T1DM mice with sodium citrate buffer, insulin, adoptively transferred nBMCs and adoptively transferred dBMCs significantly decreased the level of serum IL-10 comparing to that in naive mice received sodium citrate buffer (59.00 ± 1.39 pg/ml, 152.33 ± 5.05 pg/ml, 223.13 ± 6.75 pg/ml and 183.93 ± 7.34 pg/ml, respectively versus 262.57 ± 5.15 pg/ml) (Fig. 4G). The injection of STZ-induced T1DM mice with adoptively transferred nBMCs or adoptively transferred dBMCs significantly increased the serum level of IL-10 comparing to that in STZ-induced T1DM mice treated with insulin (223.13 ± 6.75 pg/ml and 183.93 ± 7.34 pg/ml, respectively versus 152.33 ± 5.05 pg/ml) (Fig. 4G).

### The potential of adoptively transferred BMCs against T1DM pathogenesis-related antigens and autoantibodies

**Fig. 5:**
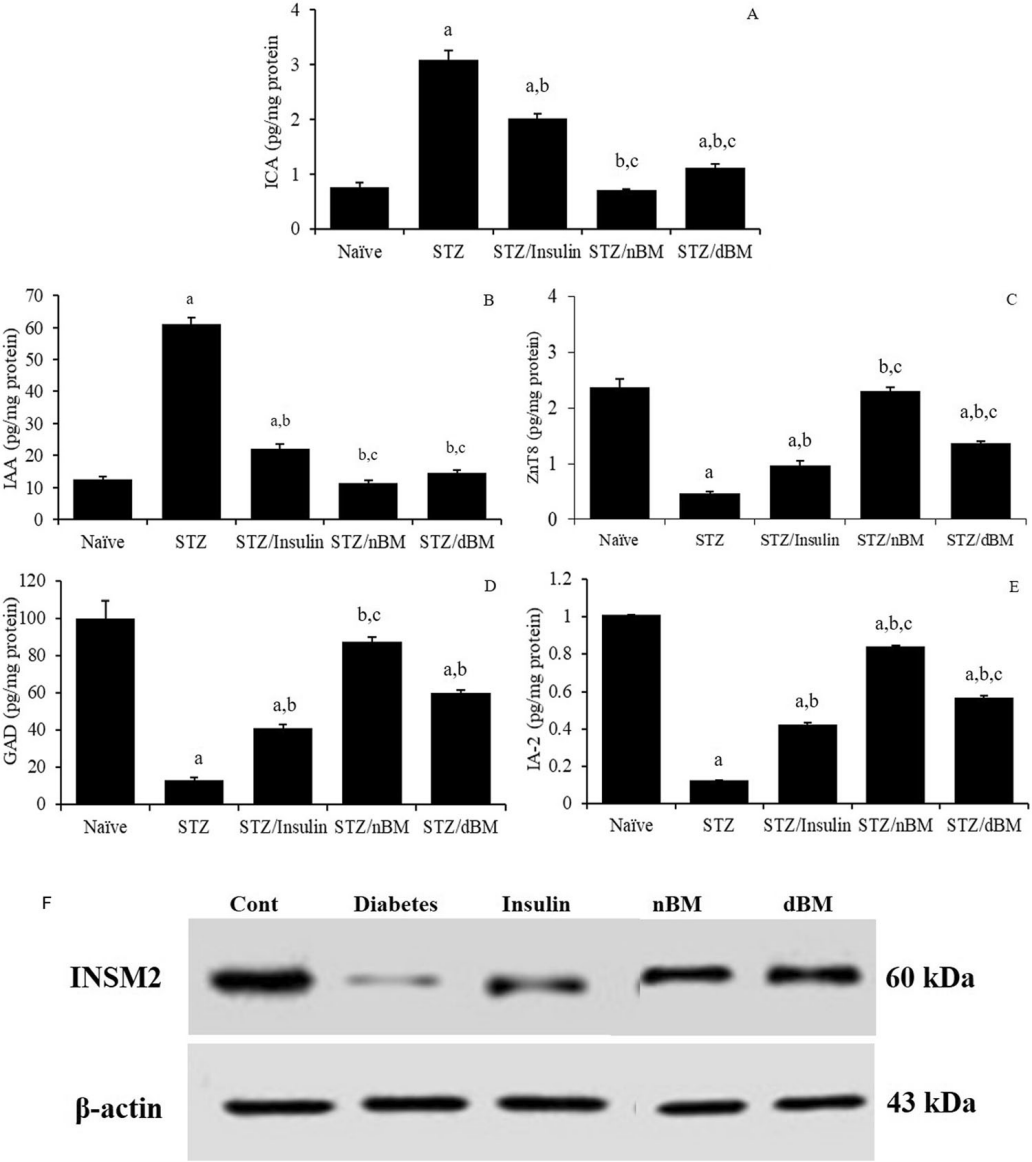
The potentials of bone marrow cells against diabetic pathogenesis-related antigens and autoantibodies in STZ-induced T1DM mice treated with adaptively transferred bone marrow-derived cells. **A** ICA level in naïve, STZ-induced T1DM mice treated with sodium citrate buffer, STZ-induced T1DM mice treated with insulin, STZ-induced T1DM mice treated with nBMCs and STZ-induced T1DM mice treated with dBMCs. **B** IAA level in naïve, STZ-induced T1DM mice treated with sodium citrate buffer, STZ-induced T1DM mice treated with insulin, STZ-induced T1DM mice treated with nBMCs and STZ-induced T1DM mice treated with dBMCs. **C** ZnT8 level in naïve, STZ-induced T1DM mice treated with sodium citrate buffer, STZ-induced T1DM mice treated with insulin, STZ-induced T1DM mice treated with nBMCs and STZ-induced T1DM mice treated with dBMCs. **D** GAD level in naïve, STZ-induced T1DM mice treated with sodium citrate buffer, STZ-induced T1DM mice treated with insulin, STZ-induced T1DM mice treated with nBMCs and STZ-induced T1DM mice treated with dBMCs. **E** IA-2 level in naïve, STZ-induced T1DM mice treated with sodium citrate buffer, STZ-induced T1DM mice treated with insulin, STZ-induced T1DM mice treated with nBMCs and STZ-induced T1DM mice treated with dBMCs. Data were expressed as mean ± SE. Statistical significant was considered as *P* value less than 0.05. Note: ^a,b,c^ Statistically significant difference as compared to the corresponding means of the naive group (a), STZ-induced T1DM mice treated with sodium citrate buffer (b), STZ-induced T1DM mice treated with insulin(c) within each column. **F** Western Blot analysis of insulinoma associated antigen 2 (INSM2) in STZ-induced T1DM mice treated with adaptively transferred bone marrow-derived cells

The current data indicated that the treatment of STZ-induced T1DM mice with sodium citrate buffer, insulin and adoptively transferred dBMCs significantly elevated the level of Islet cell autoantibodies (ICA) comparing to that in naive mice received sodium citrate buffer (3.09 ± 0.17 pg/mg, 2.01 ± 0.08 pg/mg and 1.12 ± 0.07 pg/mg, respectively versus 0.75 ± 0.0.09 pg/mg) (Fig. 5A). On the other hand, the administration of STZ-induced T1DM mice with insulin, adoptively transferred nBMCs and adoptively transferred dBMCs significantly reduced the serum level of ICA comparing to that in STZ-induced T1DM mice received sodium citrate buffer (2.01 ± 0.08 pg/mg, 0.79 ± 0.01 pg/mg and 1.12 ± 0.07 pg/mg, respectively, respectively versus 3.09 ± 0.17 pg/mg) (Fig. 5A). The inoculation of STZ-induced T1DM mice with adoptively transferred nBMCs and adoptively transferred dBMCs resulted in significant reduction in the level of ICA comparing to that in STZ-induced T1DM mice treated with insulin (0.79 ± 0.01 pg/mg and 1.12 ± 0.07 pg/mg, respectively versus 2.01 ± 0.08 pg/mg) (Fig. 5A).

Furthermore, the treatment of STZ-induced T1DM mice with sodium citrate buffer or insulin significantly elevated the level of insulin autoantibodies (IAA), comparing to that in naive mice received sodium citrate buffer (61.07 ± 1.88 pg/mg and 22.20 ± 1.40 pg/mg, respectively versus 12.63 ± 83 pg/mg) (Fig. 5B). Additionally, the treatment of STZ-induced T1DM mice with insulin, adoptively transferred nBMCs and adoptively transferred dBMCs resulted in significant decrease in the level of IAA, comparing to that in STZ-induced T1DM mice received sodium citrate buffer (22.20 ± 1.40 pg/mg, 11.20 ± 90 pg/mg and 14.67 ± 80 pg/mg, respectively, respectively versus 61.07 ± 1.88 pg/mg) (Fig. 5B). However, the treatment of STZ-induced T1DM mice with adoptively transferred nBMCs and adoptively transferred dBMCs led to significant reduction in the level of IAA, comparing to that in STZ-induced T1DM mice treated with insulin (11.20 ± 0.90 pg/mg and 14.67 ± 0.80 pg/mg, respectively versus 22.20 ± 1.40 pg/mg) (Fig. 5B).

Our data indicated that he administration of STZ-induced T1DM mice with sodium citrate buffer, insulin and adoptively transferred dBMCs significantly decreased the level of Zinc transporter 8 antigen protein (ZenT8) comparing to that in naive mice received sodium citrate buffer (0.47 ± 0.03 pg/mg, 0.97 ± 0.08 pg/mg and 1.36 ± 0.03 pg/mg, respectively versus 2.37 ± 0.14 ng/mg) (Fig. 5C). Additionally, the treatment of STZ-induced T1DM mice with insulin, adoptively transferred nBMCs and adoptively transferred dBMCs resulted in significant increase in the level of ZenT8 comparing to that in STZ-induced T1DM mice received sodium citrate buffer (0.97 ± 0.08 pg/mg, 2.30 ± 0.05 pg/mg and 1.36 ± 0.03 pg/mg, respectively, respectively versus 0.47 ± 0.05 ng/mg) (Fig. 5C). However, the treatment of STZ-induced T1DM mice with adoptively transferred nBMCs or adoptively transferred dBMCs resulted in significant increase in the level of ZenT8 comparing to that in STZ-induced T1DM mice treated with insulin (2.30 ± 0.03 pg/mg and 1.36 ± 0.03 pg/mg, respectively versus 0.97 ± 0.08 pg/mg) (Fig. 5C).

The results herein revealed that the treatment of STZ-induced T1DM mice with sodium citrate buffer, insulin and adoptively transferred dBMCs significantly decreased the level of glutamic acid decarboxylase antigen protein (GAD) comparing to that in naive mice received sodium citrate buffer (13.33 ± 1.27 pg/mg, 41.33 ± 1.76 pg/mg and 60.10 ± 1.42 pg/mg, respectively versus 100.17 ± 16.65 pg/mg) (Fig. 5D). Furthermore, the injection of STZ-induced T1DM mice with insulin, adoptively transferred nBMCs and adoptively transferred dBMCs resulted in significant increase in the level of (GAD comparing to that in STZ-induced T1DM mice received sodium citrate buffer (41.33 ± 1.76 pg/mg, 87.70 ± 2.55 pg/mg and 60.10 ± 1.42 pg/mg, respectively versus 13.33 ± 2.19 pg/mg) (Fig. 5D). The treatment of STZ-induced T1DM mice with adoptively transferred nBMCs or adoptively transferred dBMCs led to significant increase in the level of GAD comparing to that in STZ-induced T1DM mice treated with insulin (87.70 ± 2.55 pg/mg and 60.10 ± 1.42 pg/mg, respectively versus 41.33 ± 1.76 pg/mg) (Fig. 5D).

The data herein showed that the administration of STZ-induced T1DM mice with sodium citrate buffer, insulin, adoptively transferred nBMCs and adoptively transferred dBMCs significantly reduced the level of Islet antigen 2 protein (IA-2) comparing to that in naive mice received sodium citrate buffer (0.12 ± 0.01 pg/mg, 0.42 ± 0.01 pg/mg, 0.84 ± 0.01 pg/mg and 0.57 ± 0.01 pg/mg, respectively versus 1.01 ± 0.01 pg/mg) (Fig. 5E). Additionally, the treatment of STZ-induced T1DM mice with insulin, adoptively transferred nBMCs and adoptively transferred dBMCs resulted in significant elevation in the level of A-2 comparing to that in STZ-induced T1DM mice received sodium citrate buffer (0.42 ± 0.01 pg/mg, 0.84 ± 0.01 pg/mg and 0.57 ± 0.01 pg/mg, respectively versus 0.12 ± 0.01 pg/mg) (Fig. 5E). The administration of STZ-induced T1DM mice with adoptively transferred nBMCs or adoptively transferred dBMCs resulted in significant increase in the level of IA-2 comparing to that in STZ-induced T1DM mice treated with insulin (0.84 ± 0.002 pg/mg and 0.57 ± 0.01 pg/mg, respectively versus 0.42 ± 0.01 pg/mg ng/mg) (Fig. 5E).

The treatment of diabetic mice with bone marrow cells derived from naïve or diabetic mice clearly resulted in recovery of the expression of the INSM2 protein using western blotting with the INSM2 antibody which detected a single approximately 60-kDa band in mouse in pancreatic tissue comparing to those in diabetic mice received PBS or insulin. Curiously, the treatment with bone marrow cells derived from diabetic mice was more effective than bone marrow cells derived from naïve mice regarding the expression of the INSM2 protein in pancreatic tissue (Fig. 5F).

### Effect of adoptively transferred BMCs on the level of total immunoglobulin

**Fig. 6:**
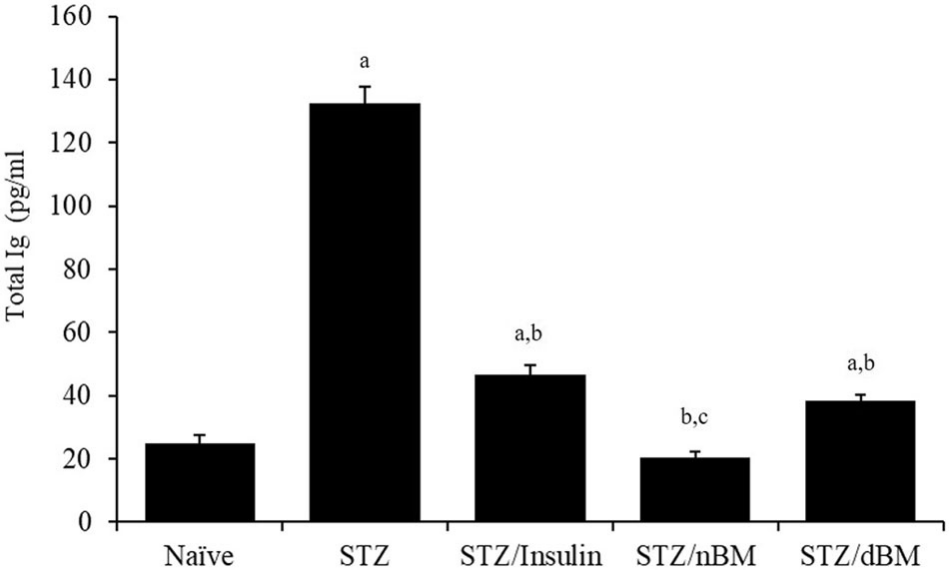
The potentials of bone marrow cells against diabetic-related immunoglobulin in STZ-induced T1DM mice treated with adaptively transferred bone marrow-derived cells. Ig level in naïve, STZ-induced T1DM mice treated with sodium citrate buffer, STZ-induced T1DM mice treated with insulin, STZ-induced T1DM mice treated with nBMCs and STZ-induced T1DM mice treated with dBMCs. Data were expressed as mean ± SE. Statistical significant was considered as P value less than 0.05. Note: a,b,c Statistically significant difference as compared to the corresponding means of the naive group (a), STZ-induced T1DM mice treated with sodium citrate buffer (b), STZ-induced T1DM mice treated with insulin(c) within each column

The administration of STZ-induced T1DM mice with sodium citrate buffer significantly increased the serum level of the total immunoglobulin (Ig), while their administration with insulin or adoptively transferred dBMCs significantly decreased the serum level of total Ig comparing to that in STZ-induced T1DM mice received sodium citrate buffer (132.33 ± 5.25 ng/ml, 46.63 ± 3.01 ng/ml and 38.56 ± 1.60 ng/ml, respectively versus 24.77 ± 2.72 ng/ml) (Fig. 6). Furthermore, the treatment of STZ-induced T1DM mice with insulin, adoptively transferred nBMCs and adoptively transferred dBMCs significantly decreased the serum level of the total Ig comparing to that in naive mice received sodium citrate buffer (46.63 ± 3.01 ng/ml, 20.33 ± 1.84 ng/ml and 38.56 ± 1.60 ng/ml, respectively versus 132.30 ± 9.11 ng/ml) (Fig. 6). The treatment of STZ-induced T1DM mice with adoptively transferred nBMCs significantly decreased the serum level of the total Ig comparing to that in STZ-induced T1DM mice treated with insulin (20.33 ± 1.84 ng/ml versus 46.63 ± 3.01 ng/ml) (Fig. 6).

### The potential of adaptively transferred BMCs on the oxidative stress and antioxidants

**Fig. 7:**
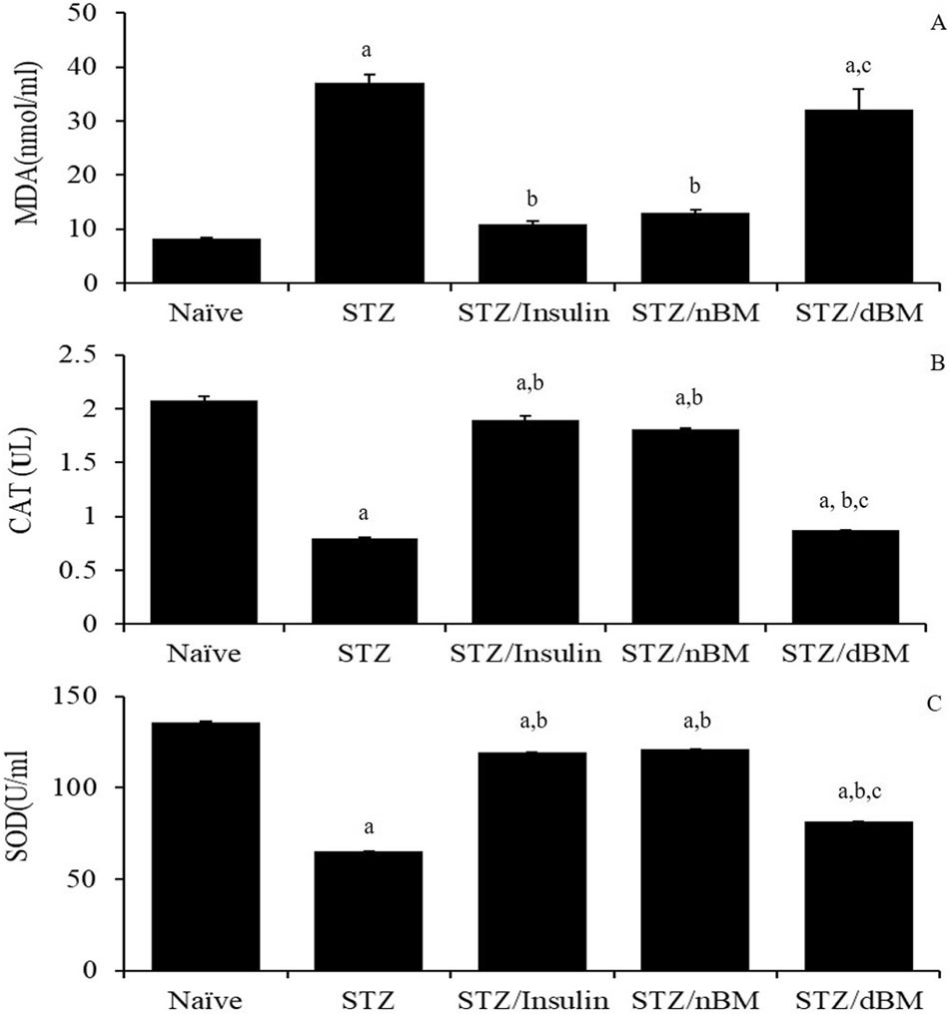
The effect of bone marrow cells on the oxidative stress and antioxidants in STZ-induced T1DM mice treated with adaptively transferred bone marrow-derived cells. **A** MDA level in naïve, STZ-induced T1DM mice treated with sodium citrate buffer, STZ-induced T1DM mice treated with insulin, STZ-induced T1DM mice treated with nBMCs and STZ-induced T1DM mice treated with dBMCs. **B** CAT level in naïve, STZ-induced T1DM mice treated with sodium citrate buffer, STZ-induced T1DM mice treated with insulin, STZ-induced T1DM mice treated with nBMCs and STZ-induced T1DM mice treated with dBMCs. **C** SOD level in naïve, STZ-induced T1DM mice treated with sodium citrate buffer, STZ-induced T1DM mice treated with insulin, STZ-induced T1DM mice treated with nBMCs and STZ-induced T1DM mice treated with dBMCs. Data were expressed as mean ± SE. Statistical significant was considered as P value less than 0.05. Note: ^a,b,c^ Statistically significant difference as compared to the corresponding means of the naive group (a), STZ-induced T1DM mice treated with sodium citrate buffer (b), STZ-induced T1DM mice treated with insulin(c) within each column

Our data herein showed that the administration of STZ-induced T1DM mice with sodium citrate buffer or adoptively transferred dBMCs significantly increased the level of serum MDA comparing to that in naive mice received sodium citrate buffer (37.10 ± 1.10 nmol/ml and 32.15 ± 2.56 nmol/ml, respectively versus 8.15 ± 0.15 nmol/ml) (Fig. 7A). Additionally, the treatment of STZ-induced T1DM mice with insulin and adoptively transferred nBMCs resulted in significant decrease in the level of serum MDA comparing to that in STZ-induced T1DM mice received sodium citrate buffer (11.00 ± 0.40 nmol/ml and 13.1 ± 0.40 nmol/ml, respectively versus 37.10 ± 1.10 nmol/ml) (Fig. 7A).

Furthermore, our data indicated that the administration of STZ-induced T1DM mice with sodium citrate buffer, insulin, adoptively transferred nBMCs or adoptively transferred dBMCs significantly decreased the level of serum CAT comparing to that in naive mice received sodium citrate buffer (0.8 ± 0.01 nmol/ml, 1.9 ± 0.03 nmol/ml, 1.81 ± 0.02 nmol/ml and 0.87 ± 0.00 nmol/ml, respectively versus 2.15 ± 0.04 nmol/ml) (Fig. 7B). Additionally, the treatment of STZ-induced T1DM mice with adoptively transferred dBMCs resulted in significant decrease in the level of serum CAT comparing to that in STZ-induced T1DM mice received sodium citrate buffer (0.87 ± 0.00 nmol/ml versus 1.9 ± 0.03 nmol/ml) (Fig. 7B).

Additionally, the data here showed that the administration of STZ-induced T1DM mice with sodium citrate buffer, insulin, adoptively transferred nBMCs or adoptively transferred dBMCs significantly decreased the level of serum SOD comparing to that in naive mice received sodium citrate buffer (65.5 ± 1.50 nmol/ml, 119.5 ± 1.50 nmol/ml, 121 ± 1.00 nmol/ml and 81.5 ± 2.50 nmol/ml, respectively versus 136.0 ± 2.00nmol/ml) (Fig. 7C). Furthermore, the administration of STZ-induced T1DM mice with insulin, adoptively transferred nBMCs and adoptively transferred dBMCs led to significant increase in the level of serum SOD comparing to that in STZ-induced T1DM mice received sodium citrate buffer (119.5 ± 2.12 nmol/ml, 121 ± 1.00 nmol/ml and 81.5 ± 2.50 nmol/ml, versus 119.5 ± 1.50 nmol/ml) (Fig. 7C). Additionally, the treatment of STZ-induced T1DM mice with adoptively transferred dBMCs resulted in significant decrease in the level of serum SOD comparing to that in STZ-induced T1DM mice received sodium citrate buffer (81.5 ± 2.50 nmol/ml versus 119.5 ± 1.50 nmol/ml) (Fig. 7C).

### Effect of adaptively transferred BMCs on the liver and kidney functions

**Fig. 8:**
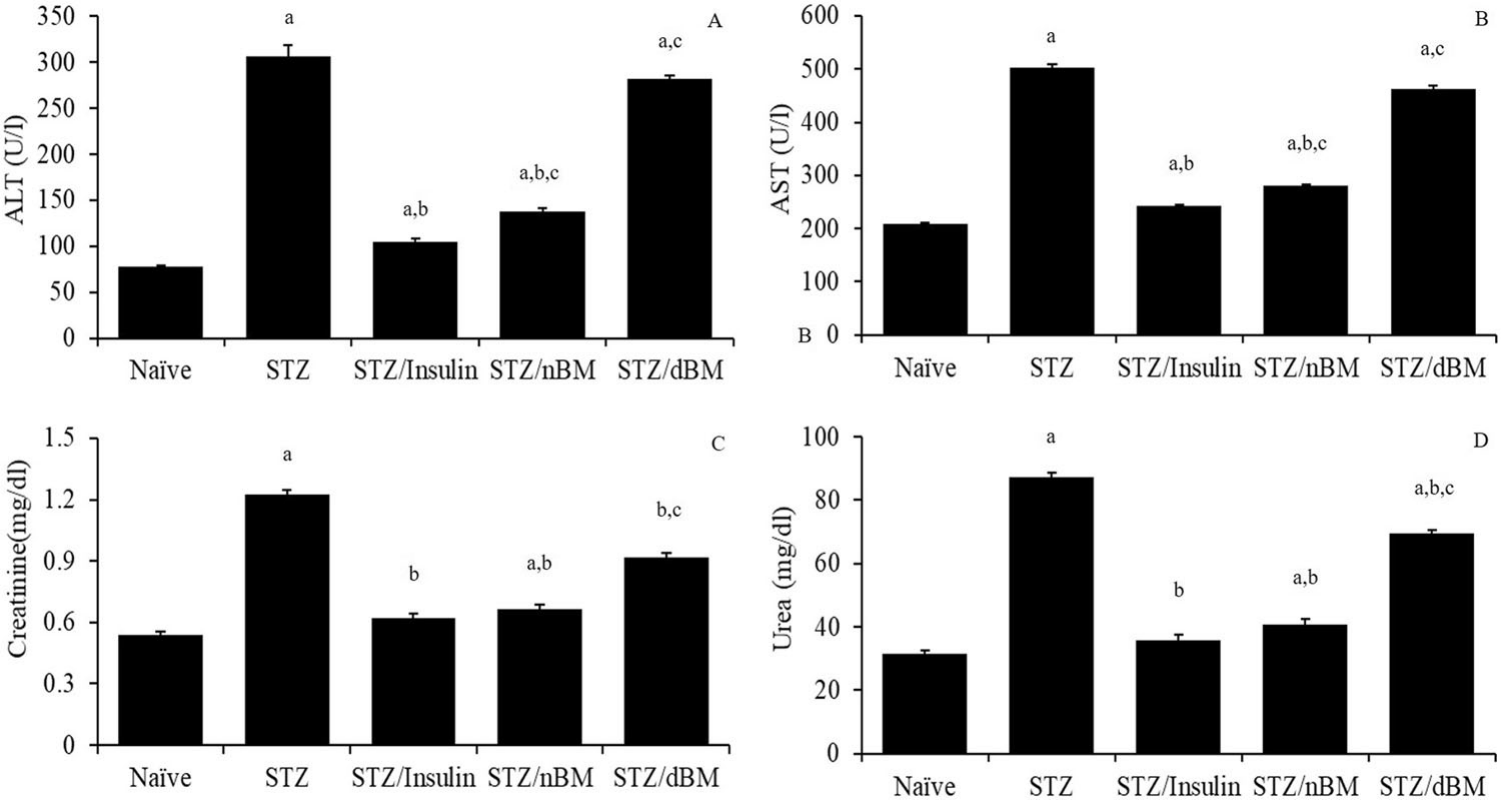
The potential of bone marrow cells on the liver and kidney functions in STZ-induced T1DM mice treated with adaptively transferred bone marrow-derived cells. **A** ALT level in naïve, STZ-induced T1DM mice treated with sodium citrate buffer, STZ-induced T1DM mice treated with insulin, STZ-induced T1DM mice treated with nBMCs and STZ-induced T1DM mice treated with dBMCs. **B** AST level in naïve, STZ-induced T1DM mice treated with sodium citrate buffer, STZ-induced T1DM mice treated with insulin, STZ-induced T1DM mice treated with nBMCs and STZ-induced T1DM mice treated with dBMCs. **C** Creatinine level in naïve, STZ-induced T1DM mice treated with sodium citrate buffer, STZ-induced T1DM mice treated with insulin, STZ-induced T1DM mice treated with nBMCs and STZ-induced T1DM mice treated with dBMCs. **D** Urea level in naïve, STZ-induced T1DM mice treated with sodium citrate buffer, STZ-induced T1DM mice treated with insulin, STZ-induced T1DM mice treated with nBMCs and STZ-induced T1DM mice treated with dBMCs. Data were expressed as mean ± SE. Statistical significant was considered as P value less than 0.05. Note: ^a,b,c^ Statistically significant difference as compared to the corresponding means of the naive group (a), STZ-induced T1DM mice treated with sodium citrate buffer (b), STZ-induced T1DM mice treated with insulin(c) within each column

Our results indicated that the treatment of STZ-induced T1DM mice with sodium citrate buffer, insulin, adoptively transferred nBMCs and adoptively transferred dBMCs significantly increased the level of serum ALT comparing to that in naive mice received sodium citrate buffer. Additionally, the treatment of STZ-induced T1DM mice with insulin or adoptively transferred nBMCs resulted in significant decrease in the level of serum ALT comparing to that in STZ-induced T1DM mice received sodium citrate buffer (Fig. 8A). The treatment of STZ-induced T1DM mice with adoptively transferred nBMCs or adoptively transferred dBMCs led to significant increase in the level of serum ALT comparing to that in STZ-induced T1DM mice received insulin (Fig. 8A).

The data herein showed that the treatment of STZ-induced T1DM mice with sodium citrate buffer, insulin, adoptively transferred nBMCs and adoptively transferred dBMCs significantly increased the level of serum AST comparing to that in naive mice received sodium citrate buffer. Additionally, the treatment of STZ-induced T1DM mice with insulin or adoptively transferred dBMCs led to significant increase in the level of serum AST comparing to that in STZ-induced T1DM mice received sodium citrate buffer (Fig. 8B). The treatment of STZ-induced T1DM mice with adoptively transferred nBMCs or adoptively transferred dBMCs led to significant increase in the level of serum ALT comparing to that in STZ-induced T1DM mice injected with insulin (Fig. 8B).

Our results showed that the administration of STZ-induced T1DM mice with sodium citrate buffer, adoptively transferred nBMCs or adoptively transferred dBMCs significantly increased the level of serum creatinine comparing to that in naive mice received sodium citrate buffer. However, the treatment of STZ-induced T1DM mice with insulin, adoptively transferred nBMCs or adoptively transferred dBMCs led to significant decrease in the level of serum creatinine comparing to that in STZ-induced T1DM mice received sodium citrate buffer (Fig. 8C), while the treatment of STZ-induced T1DM mice with adoptively transferred dBMCs result in significant increase in the level of serum creatinine comparing to that in STZ-induced T1DM mice administrated insulin (Fig. 8C).

Furthermore, our data indicated that the treatment of STZ-induced T1DM mice with insulin, adoptively transferred nBMCs and adoptively transferred dBMCs significantly elevated the level of serum urea comparing to that in naive mice received sodium citrate buffer. On the other hand, the treatment of STZ-induced T1DM mice with insulin, adoptively transferred nBMCs or adoptively transferred dBMCs resulted in significant decrease in the level of serum urea comparing to that in STZ-induced T1DM mice received sodium citrate buffer (Fig. 8D). The administration of STZ-induced T1DM mice with adoptively transferred dBMCs led to significant increase in the level of serum urea comparing to that in STZ-induced T1DM mice inoculated with insulin (Fig. 8D).

### Potential of adaptively transferred BM on the leucocyte’s indices and their differentials

**Fig. 9:**
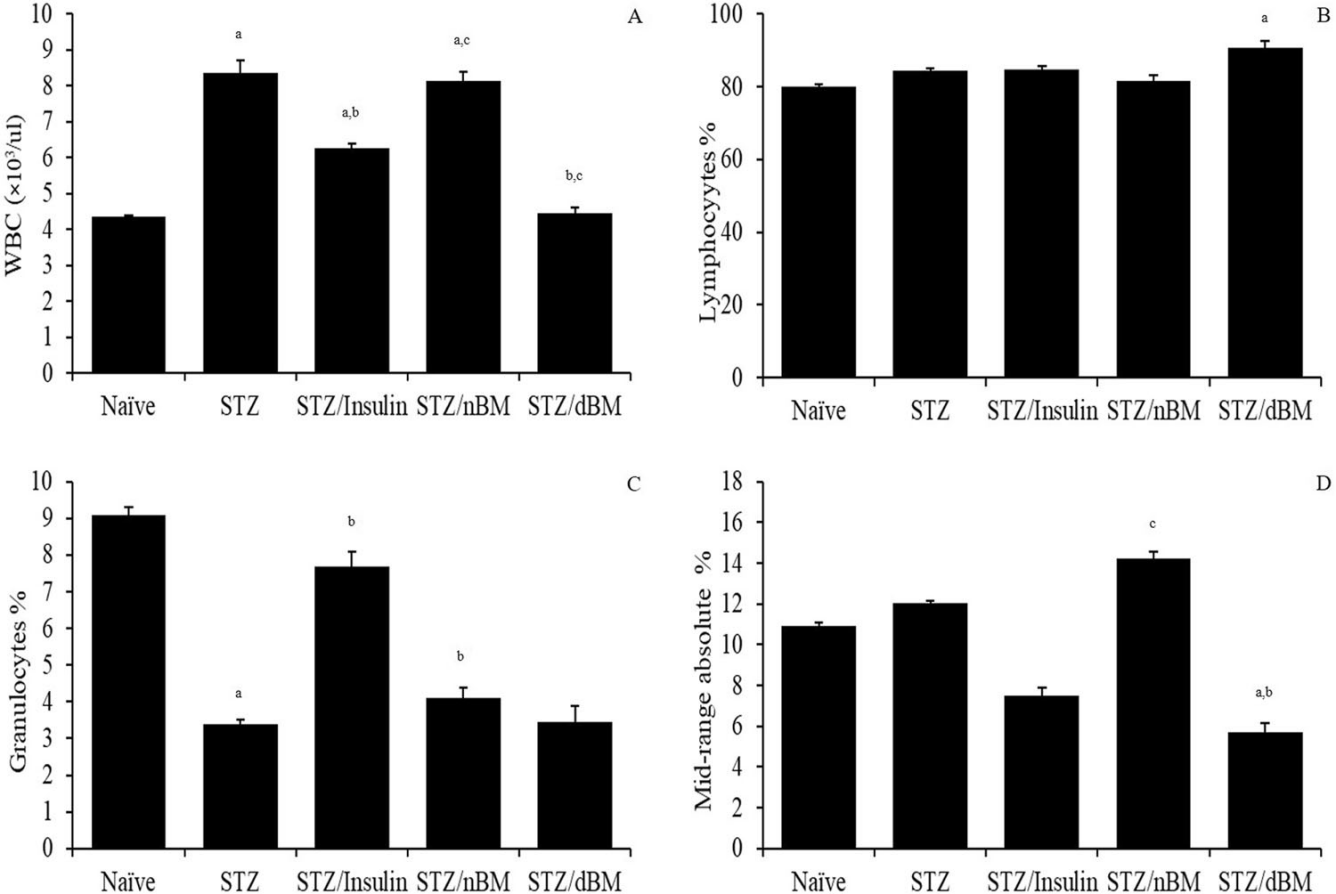
The potential of bone marrow cells on the leucocyte’s indices and their differentials in STZ-induced T1DM mice treated with adaptively transferred bone marrow-derived cells. **A** Total count of leucocytes in naïve, STZ-induced T1DM mice treated with sodium citrate buffer, STZ-induced T1DM mice treated with insulin, STZ-induced T1DM mice treated with nBMCs and STZ-induced T1DM mice treated with dBMCs. **B** Lymphocytes % in naïve, STZ-induced T1DM mice treated with sodium citrate buffer, STZ-induced T1DM mice treated with insulin, STZ-induced T1DM mice treated with nBMCs and STZ-induced T1DM mice treated with dBMCs. **C** Granulocytes % in naïve, STZ-induced T1DM mice treated with sodium citrate buffer, STZ-induced T1DM mice treated with insulin, STZ-induced T1DM mice treated with nBMCs and STZ-induced T1DM mice treated with dBMCs. **D** Mid-range absolute in naïve, STZ-induced T1DM mice treated with sodium citrate buffer, STZ-induced T1DM mice treated with insulin, STZ-induced T1DM mice treated with nBMCs and STZ-induced T1DM mice treated with dBMCs. Data were expressed as mean ± SE. Statistical significant was considered as P value less than 0.05. Note: ^a,b,c^ Statistically significant difference as compared to the corresponding means of the naive group (a), STZ-induced T1DM mice treated with sodium citrate buffer (b), STZ-induced T1DM mice treated with insulin (c) within each column

The current data showed that the administration of STZ-induced T1DM mice with sodium citrate buffer, insulin and adoptively transferred nBMCs significantly increased the total count of leucocytes comparing to that in naive mice received sodium citrate buffer (8.35 ± 0.35 × 10^3^, 6.25 ± 0.15 × 10^3^and 8.15 ± 0.25 × 10^3^, respectively versus 4.35 ± 0.05 × 10^3^) (Fig. 9A). Furthermore, the treatment of STZ-induced T1DM mice with insulin or adoptively transferred dBMCs led to significant decrease in the total count of leucocytes comparing to that in STZ-induced T1DM mice received sodium citrate buffer (6.25 ± 0.15 × 10^3^ and 4.45 ± 0.15 × 10^3^, respectively versus 8.35 ± 0.35 × 10^3^) (Fig. 9A). The treatment of STZ-induced T1DM mice with adoptively transferred nBMCs led to significant increase in the total count of leucocytes, while their treatment with adoptively transferred dBMCs resulted in significant decrease in the total count of leucocytes comparing to that in STZ-induced T1DM mice treated with insulin (8.35 ± 0.35 × 10^3^ and 4.45 ± 0.15 × 10^3^, respectively versus 6.25 ± 0.15 × 10^3^) (Fig. 9A).

The present results postulated that the treatment of STZ-induced T1DM mice with sodium citrate buffer, insulin, adoptively transferred nBMCs and adoptively transferred dBMCs led to slight increase in the relative % of lymphocytes comparing to that in naive mice received sodium citrate buffer (84.55 ± 0.55, 84.80 ± 0.90, 81.65 ± 1.65 and 90.85 ± 1.85%, respectively versus 80.00 ± 0.80%) (Fig. 9B).

Our results revealed that the treatment of STZ-induced T1DM mice with sodium citrate buffer, insulin and adoptively transferred dBMCs significantly decreased the relative % of granulocytes comparing to that in naive mice received sodium citrate buffer (3.40 ± 0.10, 4.10 ± 0.30 and 3.45 ± 0.45%, respectively versus 9.10 ± 0.20%) (Fig. 9C). Furthermore, the treatment of STZ-induced T1DM mice with insulin led to significant increase in the relative % of granulocytes comparing to that in STZ-induced T1DM mice received sodium citrate buffer (7.70 ± 40 versus 3.40 ± 0.10%) (Fig. 9C). The treatment of STZ-induced T1DM mice with adoptively transferred nBMCs and adoptively transferred dBMCs resulted in significant decrease in the relative % of granulocytes comparing to that in STZ-induced T1DM mice treated with insulin (4.10 ± 0.30 and 3.45 ± 0.45%, respectively versus 7.70 ± 0.40%) (Fig. 9C).

The administration of STZ-induced T1DM mice with adoptively transferred dBMCs significantly decreased the relative % of mid-range absolute (MID) comparing to that in naive mice received sodium citrate buffer (5.70 ± 1.40 versus 10.90 ± 0.60%) (Fig. 9D). Additionally, the administration of STZ-induced T1DM mice with adoptively transferred dBMCs resulted in significant decrease in the relative % of MID comparing to that in STZ-induced T1DM mice received insulin (5.70 ± 1.40 versus 7.50 ± 0.50%) (Fig. 9D). The treatment of STZ-induced T1DM mice with adoptively transferred nBMCs and adoptively transferred dBMCs resulted in significant decrease in the relative % of granulocytes comparing to that in STZ-induced T1DM mice treated with insulin (5.70 ± 1.40 versus 7.50 ± 0.50%) (Fig. 9D).

## Discussion

The global occurrence and frequency of T1DM are on the rise, making it a major health concern of the 21st century. Consequently, the accessibility and affordability of insulin will become challenging, particularly in underdeveloped and developing countries. Therefore, issuing alerts regarding this matter can assist international organizations and countries in strategizing preventive approaches [43]. While cell therapy is a significant treatment option for replacing damaged β-cells, its effectiveness is reduced over time by autoantibodies, resulting in the recurrence of diabetes mellitus. Therefore, there is a need for a therapeutic strategy that can recover lost insulin-producing cells by inhibiting autoimmune destruction, which is highly desirable. Bone marrow-based approaches show great promise for regeneration therapy due to their transdifferentiation potential and immunomodulatory properties [29]. The existing data demonstrated a notable rise in blood glucose and HbA1C% levels, while observing a reduction in serum insulin, in STZ-induced T1DM mice received sodium citrate buffer alone. A significant amelioration in glycemic condition has been observed due to decline in elevated blood glucose and HbA1C% levels, accompanied by an elevation in serum insulin levels in STZ-induced T1DM mice inoculated with adoptively transferred nBMCs or adoptively transferred dBMCs. It is worth emphasizing that administering adoptively transferred nBMCs or adoptively transferred dBMCs resulted in a considerable reduction in blood glucose and HbA1C% levels, ultimately restoring serum insulin levels to their initial state. These findings align with the research conducted by Akhani et al. [44] and Ahmed et al. [45], suggesting that the diabetogenic effect of STZ leads to damage and reduction in the number of pancreatic β-cells in the Langerhans islets, resulting in impaired β-cell function and reduced insulin production, ultimately leading to hyperglycemia.

This finding is consistent with the studies conducted by Ahmed et al. [1], Hu et al. [46], Tsai et al. [47], and Wang et al. [48]. It is known that bone marrow contains immature progenitor cell populations with high plasticity and the ability to differentiate into multiple cell lineages that play a role in preserving β-cells through islet protection and regeneration [49]. Additionally, BMCs can restore peripheral tolerance towards β-cells by modulating the immune response and inhibiting autoreactive T-cell function [50]. Furthermore, autologous BMT has been shown to reduce blood sugar levels and HbA1c in newly diagnosed T1DM patients [28].

The research conducted by Hess et al. [51] and revealed that blood glucose levels were significantly reduced four days after BMT into STZ-treated mice. This could be attributed to the activation of endogenous cells to produce insulin. BMCs have been discovered to enhance glycemic control in diabetic mice, albeit with temporal pattern. However, the islets were only infiltrated by bone marrow hematopoietic stem cells (HSC) and this was linked to an increased β-cell mass [13]. BMT possess immunological and regenerative properties that could be utilized to enhance the treatment of T1DM. Indeed, the utilization of SCs can potentially contribute to the restoration of peripheral tolerance towards β-cells through the alteration of the immune response and the suppression of autoreactive T-cell activity [52].

Furthermore, it has been observed that a small percentage of BMCs can undergo trans-differentiation directly into pancreatic β-cells [51, 53]. However, the reversal of hyperglycemia seems to primarily occur indirectly through increased function and/or proliferation in the remaining β-cells [54–56] or by suppressing immune responses in the damaged pancreas to facilitate endogenous regeneration [52]. Additionally, the study conducted by Arany et al. [13] revealed that the augmented β-cell mass was associated with an increase in β-cell proliferation and the number of islets, indicating that HSC may enhance both β-cell neogenesis from progenitors and proliferation in the remaining β-cells within the islets. Notably, a single intra-pancreatic injection of either bone marrow mesenchymal stem cells (BMSC) or HSC derived from mouse bone marrow can temporarily enhance glycemic control in diabetic mouse recipients, albeit with varying time courses and likely different mechanisms of action. Notably, BM-MSCs have demonstrated therapeutic effects in improving glycemic control, blood glucose levels, and preserving β-cell function in T1D [57].

BMCs possess the remarkable ability to enhance the size of islets by differentiating into β-cell-like organoids. Additionally, they can restore immunotolerance by suppressing the immune response of T cells and Th1 cells through the activation of TGF-β and inflammatory mechanisms [11]. Given that T1DM is characterized as an autoimmune disease, where immune cells attack and destroy pancreatic β -cells, it is crucial to consider the immunomodulatory properties of BMCs and their ability to differentiate into insulin-producing cells when utilizing BMT for T1DM treatment. Notably, HSCs have demonstrated the ability to inhibit the development of T1DM [58, 59]. HSCs, with their immunomodulatory properties, offer a potent immunoregulatory effect without depleting T lymphocytes. In fact, HSCs can reset the immune response, thereby restoring a more self-tolerant state in the chronically disrupted immune system [60]. To prevent the development of experimental autoimmune diabetes in murine models, various approaches have been taken that rely on HSCs to reinstate the elimination of autoreactive T cells in the peripheral system. The infusion of HSCs has proven highly successful in preventing diabetes onset in non-obese diabetic (NOD) mice through the induction of mixed chimerism [61]. Additionally, immune tolerance can be restored in the peripheral tissues. BMCs possess both regenerative and immunological properties, making them a potential tool for improving the treatment of T1DM. By reshaping the immune response and inhibiting the function of autoreactive T cells, BMCs may help reestablish peripheral tolerance towards β-cells. Moreover, insulin-producing cells derived from bone marrow stem cells have shown the ability to engraft and reverse hyperglycemia in mice. Bone marrow mesenchymal stem cells exhibit a hypoimmunogenic phenotype and possess a wide range of immunomodulatory capabilities [52]. The enhancement of β-cell mass and function can involve the fusion of BMSC with islet cells or the trans-differentiation of these stem cells into β-cells. Furthermore, their immunomodulatory functions can be influenced by the local environment [62, 63].

BMT has the potential to revolutionize the treatment of diabetes by producing functional insulin-secreting β-cells. The regenerative and immunomodulatory abilities of these BMCs highlight their therapeutic potential. In a study by Farooq et al. [64], BMCs were found to reduce hyperglycemia when transferred into chemically induced hyperglycemic mice. These cells were mainly localized to ductal and islet structures, leading to an increase in insulin production. Bone marrow HSCs, as observed by Hasegawa et al. [23], can promote the regeneration of β-cells rather than directly differentiating or through their immunological properties. Kang et al. [65] successfully prevented the onset of T1DM in mice by transplanting allogeneic HSCs, although reversal was achieved in only one out of 50 mice. Mishra et al. [66] discovered that insulin-producing cells (IPCs) can be derived from various BMCs, such as adult, mesenchymal, and hematopoietic stem cells, through processes like proliferation, dedifferentiation, neogenesis, nuclear reprogramming, and trans-differentiation. Overall, these findings demonstrate the promising potential of bone marrow cells-based strategies in the treatment of diabetes.

The current data indicate that administering adoptively transferred nBMCs or adoptively transferred dBMCs led to a decrease in levels of inflammatory cytokine markers in the serum, including INF-γ, TNF-α, TGF-β, IL-1β, IL-4, IL-6, and IL-10. These findings agree with Izadi et al. [67] study that revealed the positive effects of early transplantation of MSCs on metabolic indices and immune responses. They found that early transplantation resulted in an increase in anti-inflammatory cytokines and a decrease in pro-inflammatory cytokines compared to late transplantation. This was evident through a significant rise in the level of serum IL-10, an important anti-inflammatory cytokine, and a slight increase in TGF-β1 after 12 months. Additionally, there was a notable reduction in serum TNF-α and a moderate decrease in IL-6 levels at 12 months, both of which are pro-inflammatory cytokines. Previous studies have also shown a decrease in IL-4 levels in newly diagnosed T1D patients. IL-10 plays an essential role in mediating immunosuppressive functions. Specifically, only IL-10 + B cells, not whole B-cell populations or IL-10 − B-cell subsets, were able to suppress autoreactive T-cell activation ex vivo and prevent the diabetes progression in vivo. Within islets, there were increased levels of regulatory B (Breg)-enhancing CD40 + B cells and IL-10–producing B cells, which provided protection against autoimmune destruction of pancreatic islets and had the necessary requirements for maintenance and expansion in vivo [68]. Furthermore, there is a close correlation between rised IL-10 levels and disease attenuation. Preserving β-cell function in newly diagnosed T1D patients can be achieved through the administration of TNF blockers [69] or an IL-6 blockade [70]. The use of MSCs in transplantation has been shown to shift the balance of cytokines in the blood from pro-inflammatory to anti-inflammatory, increase the number of Treg cells in the peripheral blood, and improve overall quality of life [67]. IL-10 plays an essential role in modulating the inflammatory response by inhibiting the functional activity of Th1 cells and various inflammatory interleukins, including TNF-α, IL-1β, and IL-6. Additionally, it promotes cell proliferation in B lymphocytes while preventing excessive cell proliferation and apoptosis. The effects of IL-10 extend beyond its anti-inflammatory properties, encompassing the inhibition of Th1 cells, pro-inflammatory cytokines (TNF-α, IL-1β, IL-6), and antigen-presenting cells. It also stimulates tissue repair mechanisms and plays a vital role in restraining an exaggerated inflammatory response [71]. Furthermore, IL-10 has been associated with the induction of a tolerant state in immature dendritic cells and the release of regulatory IL-10-producing B cells in individuals with T1DM [68, 72].

Notably, IL-10 enhances the anti-apoptotic and anti-inflammatory capabilities of islets without affecting systemic immunity [73]. MSCs have the potential to inhibit the differentiation of monocytes, the maturation of DCs, and the proliferation and activation of B cells, cytotoxic T cells, and NK cells. At the same time, they promote the generation and activation of Breg cells and Treg cells [74]. In various murine models, different subsets of activated B cells have been known to suppress the proliferation of antigen-specific CD4 + T cells and the production of pro-inflammatory cytokines in an IL-10-dependent manner. This suppression of inflammation has been observed in vitro and in vivo in autoimmune conditions like type 1 diabetes [75, 76]. MSCs also express interleukin-10 constitutively, which plays a well-established role in regulating T cells and promoting a suppressor phenotype by counteracting the effects of IL-12 during the initiation of inflammatory immune responses. Additionally, the protective effects of IFN-γ are associated with the inhibition of diabetogenic CD8 + T cell responses by reducing the signal transducer and activator of transcription (STAT-1) protein and decreasing the levels of Th1 cell-related cytokines, particularly IL-12 and IL-2, in spleen cells [77].

The recent study demonstrated that STZ-induced T1DM mice, when treated with adoptively transferred nBMCs or adoptively transferred dBMCs, experienced a notable rise in total immunoglobulin (Ig) levels. Additionally, there was a significant reduction in the levels of Islet cell autoantibodies (ICA) and insulin autoantibodies (IAA). Furthermore, the serum of STZ-induced T1DM mice, treated with adoptively transferred nBMCs or adoptively transferred dBMCs, showed a significant increase in Zinc transporter 8 antigen protein (ZnT8), Islet antigen 2 protein (IA-2), and glutamic acid decarboxylase antigen protein (GAD) levels. Interestingly, the administration of nBMCs or dBMCs resulted in a heightened expression of IA-2 protein. IA-2 plays a regulatory role in the mobilization and recruitment of secretary vesicles during glucose-stimulated insulin secretion [78]. Additionally, the elimination of IA-2 and/or IA-2β leads to a decline in insulin levels and release [34]. Moreover, znt8 is a unique protein found only in islets and plays a crucial role in transferring zinc from the cytoplasm to secretory vesicles. This protein regulates the concentration of zinc in β cells and acts as a zinc sensor.

Consequently, ZnT8 plays a crucial role in regulating glucose homeostasis [35]. Our findings align with previous studies by Gianani et al. [38] and Sue et al. [39], which reported that autoimmune diabetes is characterized by the presence of anti-islet antibodies (ICA), anti-glutamic acid decarboxylase antibodies (anti-GAD antibodies), and anti-insulin antibodies, all of which are strongly associated with the development of T1DM. The specificity of autoantibodies against pancreatic islet cells (ICA), IA-2, and insulin antibodies is particularly high in autoimmune diabetes. On the other hand, treating STZ-induced T1DM mice with bone marrow cells derived from either naïve or diabetic mice led to an increase in serum levels of ZnT8, IA-2, and GAD protein, while decreasing total immunoglobulin (Ig), IAA, and ICA autoantibody levels. These findings align with the results obtained by Mesples et al. [28], who demonstrated that the levels of ICA, GAD, and IA2 antibodies were reduced in newly diagnosed T1D patients following autologous BMT, thereby suppressing autoimmune attack and pancreatic damage. Furthermore, treatment with autologous BMCs resulted in a negative value for ICA, GAD, and anti-insulin antibody levels, which persisted during the 12-month follow-up period, effectively reversing the production and impact of anti-pancreatic islet antibodies [28]. It is worth noting that Insm2 plays a crucial role in glucose-stimulated insulin secretion [79].

In our research, we observed that the levels of MDA were increased in STZ-induced T1DM mice, while the levels of CAT and SOD were decreased. However, when adoptively transferred nBMCs or adoptively transferred dBMCs were administered to STZ-induced T1DM mice, it had a significant impact on reducing oxidative stress. This was accomplished by reducing the levels of MDA in the serum and enhancing the activities of enzymatic antioxidants like CAT and SOD. These results are in line with the study carried out by Refat et al. [80] indicating that diabetes is associated with elevated MDA levels and decreased antioxidant enzyme levels (SOD, CAT, GRx, and GST). Oxidative stress plays a significant role in the progression of both microvascular and cardiovascular complications associated with diabetes. The metabolic abnormalities associated with diabetes lead to the overproduction of mitochondrial superoxide in endothelial cells of both large and small vessels [80]. Permanent hyperglycemia may promote free radical production through auto-oxidation of lipid peroxides and dysfunction of the antioxidant defense system, leading to increased free radical levels and oxidative stress [81]. This may be due to a link between chronic inflammatory states and impaired insulin activity through the stimulation of insulin receptor expression on β-cells, as suggested by Targher et al. [82].

These bone marrow cells were able to regenerate the damaged β-cells and improve the glycemic state. The increase in antioxidant activity can be attributed to the ability of the bone marrow cells to prevent the formation of free radicals. This improvement in endogenous antioxidant activity goes beyond their capacity to scavenge free radicals and leads to a decrease in hepatic lipoperoxide formation. The treatment with bone marrow cells not only suppresses oxidative stress but also enhances the antioxidant defense system. This plays a significant role in improving the function of pancreatic islets, resulting in an increase in blood insulin levels and an overall improvement in the glycemic state. These findings align with the research conducted by Stavely and Nurgali [83], who also demonstrated the importance of enzymatic antioxidants and nonenzymatic antioxidants in regulating redox homeostasis and preventing excessive production of free radicals. It is believed that MSCs possess anti-oxidant potential through direct scavenging of reactive oxygen species (ROS), donation of mitochondria, or indirect upregulation of antioxidant defenses in other cells. This, in turn, leads to cytoprotective and anti-inflammatory effects. Additionally, MSCs are associated with the expression of constitutively expressed antioxidant enzymes such as SOD and CAT, which further modulate the inflammatory response and enhance cellular respiration and mitochondrial functions. They may also donate their mitochondria to protect damaged cells [84, 85].

In the present study, STZ-induced T1DM mice displayed a significant elevation in the levels of liver enzymes ALT and AST, as well as heightened levels of creatinine and urea. Considering the crucial roles of the liver and kidney in metabolism and excretion, this research further examined the effects of administering adoptively transferred nBMCs or adoptively transferred dBMCs to STZ-induced T1DM mice. Notably, the administration of these cells alleviated the observed effects. These findings align with the study conducted by Shady et al. [86], which demonstrated that different types of MSCs have protective effects on the liver and kidneys, as evidenced by the decreased levels of markers for liver and kidney function in treated diabetic rats. Overall, our results emphasize the therapeutic advantages of BMT in alleviating metabolic abnormalities and diabetic complications in the liver and kidneys. Additionally, BMCs contribute to these effects through different mechanisms such as mitosis, neovascularization, anti-inflammatory actions, cytoprotection, inhibition of excessive production, and immune regulation [87].

BMT has the potential to alleviate chronic kidney damage, including STZ-induced diabetic nephropathy, through homing and differentiation. This process allows the bone marrow stem cells to recognize damaged tissues, integrate into specific sites, and differentiate into renal tissue cells in specific environmental conditions [88–90]. Additionally, BMT has shown promising results in initiating significant renal function recovery in diabetic rats, as evidenced by the decline in serum uric acid, urea, and creatinine levels [91, 92]. The therapeutic potential of MSCs mainly involves a paracrine mechanism, which includes modulation of the immune response, extracellular vesicles and trophic factors release, anti-fibrotic effects, and renal tissue repair [88]. Furthermore, MSCs infusion at the early stages of diabetic nephropathy has been found to suppress renal cytokine and macrophage infiltration, inhibit nephrocyte apoptosis, and attenuate renal impairment in diabetic rats, thereby preventing glomerular defects and kidney dysfunction [93].

Moreover, studies have demonstrated that BM-MSCs therapy can enhance the natural regenerative processes of hepatocytes in patients with advanced chronic liver disease. This is due to the unique self-renewal and differentiation capabilities of BM-MSCs, which can ameliorate liver function. Studies have also demonstrated that BM-MSCs have protective abilities on both the structure and function of hepatocytes in STZ-diabetic rats, as evidenced by the significant improvement in serum levels of total bilirubin, ALP, ALT, AST and other markers [94]. Additionally, BM-MSCs have been shown to protect against liver fibrosis and promote the recovery of injured liver function, as shown by reduced serum levels of AST, ALT, ALP, total bilirubin, and improved histological lesions of liver tissue. These findings suggest that MSCs transplantation may be an effective therapeutic approach for T2DM patients with liver fibrosis [91, 95]. Furthermore, MSCs can release paracrine factors that modulate the immune response, stimulate cell proliferation, restore liver function, and attenuate cell death mechanisms. These factors collectively support the hepatocyte differentiation potential of MSCs and highlight their potential hepato-protective role in DM, which could enhance liver function and restore appropriate protein metabolism balance [96].

## Conclusion

To summarize, the administration of bone marrow cells derived from either nBMCs or dBMCs has shown significant anti-hyperglycemic effects in STZ-induced T1DM mice. Notably, nBMCs proved to be the most effective. These anti-hyperglycemic effects are likely attributed to improvements in the function of β-cells, resulting in increased insulin secretion and reduced HbA1C production. The suppressive effects of BMCs on inflammation and oxidative stress, along with their immunomodulatory and their ability to enhance the anti-oxidant defense system, may be responsible for the beneficial influence on the structure of pancreatic islets and the integrity of β-cells. Nevertheless, additional investigation is required to explore the molecular mechanisms and pathways that underlie the impact of BMCs in the management of D1MT. Furthermore, it is imperative to conduct clinical studies to assess the efficacy and safety of utilizing BMCs in diabetic patients prior to considering them as a prospective therapy for T1DM.

## Data Availability

The data that support the findings of this study are available from the corresponding author upon reasonable request.
